# Transcriptional maturation of the mouse auditory forebrain

**DOI:** 10.1186/s12864-015-1709-8

**Published:** 2015-08-14

**Authors:** Troy A. Hackett, Yan Guo, Amanda Clause, Nicholas J. Hackett, Krassimira Garbett, Pan Zhang, Daniel B. Polley, Karoly Mirnics

**Affiliations:** Department of Hearing and Speech Sciences, Vanderbilt University School of Medicine, Nashville, TN USA; Eaton-Peabody Laboratories, Massachusetts Eye and Ear Infirmary, Department of Otology and Laryngology, Harvard Medical School, Boston, MA USA; Northwestern University Feinberg School of Medicine, Chicago, IL USA; Department of Cancer Biology, Vanderbilt University, Nashville, TN USA; Department of Psychiatry, Vanderbilt University, Nashville, TN USA; Vanderbilt Institute for Integrative Biosystems Research and Education, Vanderbilt University, Nashville, TN 37235 USA; Department of Psychiatry, University of Szeged, 6725 Szeged, Hungary; Vanderbilt Kennedy Center for Research on Human Development, Vanderbilt University, Nashville, TN 37232 USA

**Keywords:** Synapse, Plasticity, Development, Critical period, Cortex, Thalamus, Neurotransmission, Neuromodulation, Extracellular matrix, Myelination, RNAseq, Pathway analysis, Sequencing, RNA

## Abstract

**Background:**

The maturation of the brain involves the coordinated expression of thousands of genes, proteins and regulatory elements over time. In sensory pathways, gene expression profiles are modified by age and sensory experience in a manner that differs between brain regions and cell types. In the auditory system of altricial animals, neuronal activity increases markedly after the opening of the ear canals, initiating events that culminate in the maturation of auditory circuitry in the brain. This window provides a unique opportunity to study how gene expression patterns are modified by the onset of sensory experience through maturity. As a tool for capturing these features, next-generation sequencing of total RNA (RNAseq) has tremendous utility, because the entire transcriptome can be screened to index expression of any gene. To date, whole transcriptome profiles have not been generated for any central auditory structure in any species at any age. In the present study, RNAseq was used to profile two regions of the mouse auditory forebrain (A1, primary auditory cortex; MG, medial geniculate) at key stages of postnatal development (P7, P14, P21, adult) before and after the onset of hearing (~P12). Hierarchical clustering, differential expression, and functional geneset enrichment analyses (GSEA) were used to profile the expression patterns of all genes. Selected genesets related to neurotransmission, developmental plasticity, critical periods and brain structure were highlighted. An accessible repository of the entire dataset was also constructed that permits extraction and screening of all data from the global through single-gene levels. To our knowledge, this is the first whole transcriptome sequencing study of the forebrain of any mammalian sensory system. Although the data are most relevant for the auditory system, they are generally applicable to forebrain structures in the visual and somatosensory systems, as well.

**Results:**

The main findings were: (1) Global gene expression patterns were tightly clustered by postnatal age and brain region; (2) comparing A1 and MG, the total numbers of differentially expressed genes were comparable from P7 to P21, then dropped to nearly half by adulthood; (3) comparing successive age groups, the greatest numbers of differentially expressed genes were found between P7 and P14 in both regions, followed by a steady decline in numbers with age; (4) maturational trajectories in expression levels varied at the single gene level (increasing, decreasing, static, other); (5) between regions, the profiles of single genes were often asymmetric; (6) GSEA revealed that genesets related to neural activity and plasticity were typically upregulated from P7 to adult, while those related to structure tended to be downregulated; (7) GSEA and pathways analysis of selected functional networks were not predictive of expression patterns in the auditory forebrain for all genes, reflecting regional specificity at the single gene level.

**Conclusions:**

Gene expression in the auditory forebrain during postnatal development is in constant flux and becomes increasingly stable with age. Maturational changes are evident at the global through single gene levels. Transcriptome profiles in A1 and MG are distinct at all ages, and differ from other brain regions. The database generated by this study provides a rich foundation for the identification of novel developmental biomarkers, functional gene pathways, and targeted studies of postnatal maturation in the auditory forebrain.

**Electronic supplementary material:**

The online version of this article (doi:10.1186/s12864-015-1709-8) contains supplementary material, which is available to authorized users.

## Background

The development and maturation of the brain is an exceptionally complex biological process that depends on the coordinated expression of many thousands of genes and proteins [1]. In every region of the brain, much remains to be learned about the spatial and temporal properties of their expression patterns, regulation, and functional roles.

An important goal in sensory systems research is to understand the mechanisms that govern maturation, and how these factors affect or are affected by major milestones, such as the emergence of intrinsically generated electrical signals or the onset of activity evoked by extrinsic stimuli from the sensory environment. In the central auditory pathways of rodents, structural and functional development begins during gestation and continues through the first three to four postnatal weeks. Auditory processing capabilities develop rapidly between postnatal days P10–P16, catalyzed by the opening of the ear canals (~P12) and the associated shift from intrinsically to extrinsically generated patterns of electrical activity [2–5]. This window has provided researchers with a unique opportunity to document structural and functional maturation associated with the onset of hearing [6–9], and the formation of critical periods for the plasticity of sound feature encoding and behavior [10–14]. In this context, *plasticity* refers to the potential for structural and functional change at the level of the synapse or networks of neurons. These changes are mediated by intrinsic mechanisms at the cellular and molecular levels, and shaped by extrinsic factors, such as the onset of sensory experience. *Critical periods* are windows of time during which the conditions for plasticity are such that the functional properties of a synapse or network can be altered by experience (or lack, thereof) in a manner that has long-lasting or permanent effects [15].

Efforts to characterize the cellular and molecular landscape during maturation, and their relationship to the specific mechanisms that regulate plasticity and critical periods are ongoing. A wide range of factors has been explored. Synaptic inhibition (GABA) and excitation (glutamate) are considered to be central regulators in the maturation of auditory response properties, and continue to be intensively studied [16–20]. Other studies have focused on the influences of neuromodulatory inputs (e.g., cholinergic, dopaminergic, serotonergic) [21–26] and the roles played by ion channels [27]. Myriad structural factors also impact neuronal activity, such as dendritic spine formation [28], gap junctions [29], synaptic morphology [30, 31], and myelin signaling and extracellular matrix formation [32, 14].

These studies, and many more, have contributed much to our understanding of the mechanisms involved. Yet, much remains to be learned, and it may be that important, even essential, mechanisms have not yet been identified. Lacking so far is application of a comprehensive broad-spectrum approach to identify novel mechanisms on a large scale. Among the techniques that could be employed, whole transcriptome sequencing of total RNA is a powerful tool for the generation of gene expression profiles and identification of functional biomarkers. By sequencing samples from different brain regions at several time-points during development, a series of snapshots documenting the influences of age and experience on the entire transcriptome can be acquired. This permits identification of significant changes in the expression of any coding or non-coding gene. So far, targeted profiling of up to about 2000 genes or proteins has been successfully used to identify changes in auditory brainstem nuclei associated with postnatal development [33, 34], hearing loss [35], and auditory cortex lesions [36]. To date, however, whole transcriptome profiles have not been generated for any central auditory structure in any species at any age.

To enhance the foundation for discovery along these lines, we used high-throughput next-generation sequencing of total RNA (RNAseq) to profile RNA expression in the primary auditory cortex (A1) and medial geniculate body (MG) of mice at selected time-points during postnatal development, before and after the onset of hearing (postnatal days P7, P14, P21, and adult). Differential expression analyses were employed to compare the transcriptomes between brain regions and age groups. Functional gene set analyses were performed to create reference libraries of gene families and functional gene ontology categories that have importance for brain function and developmental neurobiology. Several representative genesets were profiled in detail at the single gene level, one of which was explored by pathways analysis. Finally, all of the data were organized into an accessible and searchable database that facilitates the identification of genes that are involved in the maturation of the auditory forebrain.

To our knowledge, this is the first whole transcriptome sequencing study of maturation in the forebrain of any mammalian sensory system. Although the data are most relevant for the auditory system, they are generally applicable to forebrain structures in the visual and somatosensory systems, as well.

## Methods

### Tissue acquisition

All procedures were approved by the Animal Care and Use Committee at Massachusetts Eye and Ear Infirmary and followed the guidelines established by the National Institutes of Health for the care and use of laboratory animals. The morning that a new litter of pups was first observed was designated as P0. Brains were collected from 24 adult (8–10 weeks) and juvenile (P7, P14, and P21) male and female C57BL/6 J mice (Jackson Labs 000664) (*N* = 6 per age, equal numbers of males and females, total = 24). Animals were euthanized with a lethal dose of ketamine and xylazine (200/50 mg/kg, respectively) intraperitoneally. Brains were removed immediately, flash frozen on dry ice, and stored at−80^0^ C.

### Sample acquisition

Frozen brains from 6 animals in each age group (3 male, 3 female) were sectioned at 40 μm in the coronal plane (rostral to caudal) on a sliding microtome and viewed through a surgical microscope. As established anatomical landmarks [37] became visible in the frozen tissue block, the regions targeted for sampling (A1, primary auditory cortex; MG, medial geniculate body), were extracted using a sterile tissue punch or curette of a size appropriate to the brain region (Additional file 1: Figure S1) (note that Additional Figs and Tables are indicated by inclusion of the letter S before the number). Punches from homologous areas of both hemispheres were combined in sterile tubes containing 400 μl of Trizol, homogenized for 45 s using a mechanized sterile pestle, flash frozen on dry ice, then stored at−80^0^ C. Each of the A1 and MG sample pairs were from the same animals, as indicated by the Sample ID and Animal ID codes in Additional file 2: Table S1.

A1 samples were obtained using a 0.5 mm diameter punch, with the ventral edge beginning approximately 1 mm dorsal to the rhinal fissure. Samples were centered on A1, but potentially also included some tissue in the adjacent auditory field dorsal to A1. MG samples were harvested with a curette after using a micro-dissecting scalpel to circumscribe its perimeter (Additional file 1: Figure S1b). For the MG, the microdissection procedure was intended to exclude the lateral geniculate nucleus (LGN), which was achieved by identification of the septum between the MG and LGN dorsolaterally, in the rostral third of the MG. Because there are no remnants of the LGN caudal to the MG at this point, the LGN was easily excluded by the dissection. Additional evidence that the LGN was successfully excluded is supported by comparison of our results with a prior study comparing the expression of a subset of genes in LGN and MG [38]. In that study, 10 genes had moderately-high to high levels of expression in the LGN (*Zic4, Zic5, Ecel1, Isl1, Npy, Arx, Pvalb, Pmch, Pax6, Zfp503*). All of these genes had very low to nominal expression at all age in the MG of our samples. Therefore, we conclude that there was no significant contamination by LGN. The dissection was also intended to exclude adjoining nuclei located medial, and ventral to the MG, but some tissue from these nuclei may have been included (e.g., suprageniculate, peripeduncular). The extreme rostral and caudal poles of the MG were largely excluded from these samples.

### RNA extraction and sequencing

For each Trizol lysate, 100 μl of Reagent Grade Chloroform (Fisher Scientific, S25248) was added. The samples were centrifuged for 3 min on a desktop centrifuge to fractionate the aqueous and organic layers. After centrifugation, the resulting aqueous layer was carefully removed and transferred to 2.0 ml Sarstedt tubes (Sarstedt, 72.694) which were run on the QIAsymphony SP (Qiagen Corporation, Germany) using the QIAsymphony RNA Kit (Qiagen, 931636) and protocol RNA_CT_400_V7 which incorporates DNAse treatment. Prior to each run, the desk was uv-irradiated using the programmed cycle. The resulting RNA was eluted to 100 μl of RNase free water and stored at−80 °C in 2.0 ml Sarstedt tubes until use. Samples were initially quantitated using a Qubit fluorometric RNA assay (Life Technologies, Grand Island, NY). Additional analyses of purity and the quantitation of total RNA were performed using a NanoDrop spectrophotometer (Thermo Scientific) and Agilent RNA 6000 Pico chip (Agilent) according to the manufacturer’s protocol using the reagents, chips, and ladder provided in the kit. Quality control data for the 48 sequenced samples are contained in Additional file 2: Table S1.

RNAseq was performed by the Vanderbilt Technologies for Advanced Genomics core (VANTAGE). First, ribosomal reduction was performed on 1 μg total RNA using the Ribo-Zero Magnetic Gold Kit (Human/Mouse/Rat) (Epicentre), following the manufacturer’s protocol. After ribosomal RNA (rRNA) depletion, samples were purified using the Agencourt RNAClean XP Kit (Beckman Coulter) according to the Epicentre protocol specifications. After purification, samples were eluted in 11 μl RNase-free water. Next, 1ul ribosomal depleted samples were run on the Agilent RNA 6000 Pico Chip to confirm rRNA removal. After confirmation of rRNA removal, 8.5 μl rRNA-depleted sample was input into the Illumina TruSeq Stranded RNA Sample Preparation kit (Illumina) for library preparation. Libraries were multiplexed six per lane and sequenced on the HiSeq 2500 to obtain at least 30 million paired end (2x50 bp) reads per sample.

### RNAseq data processing

**Fig. 1:**
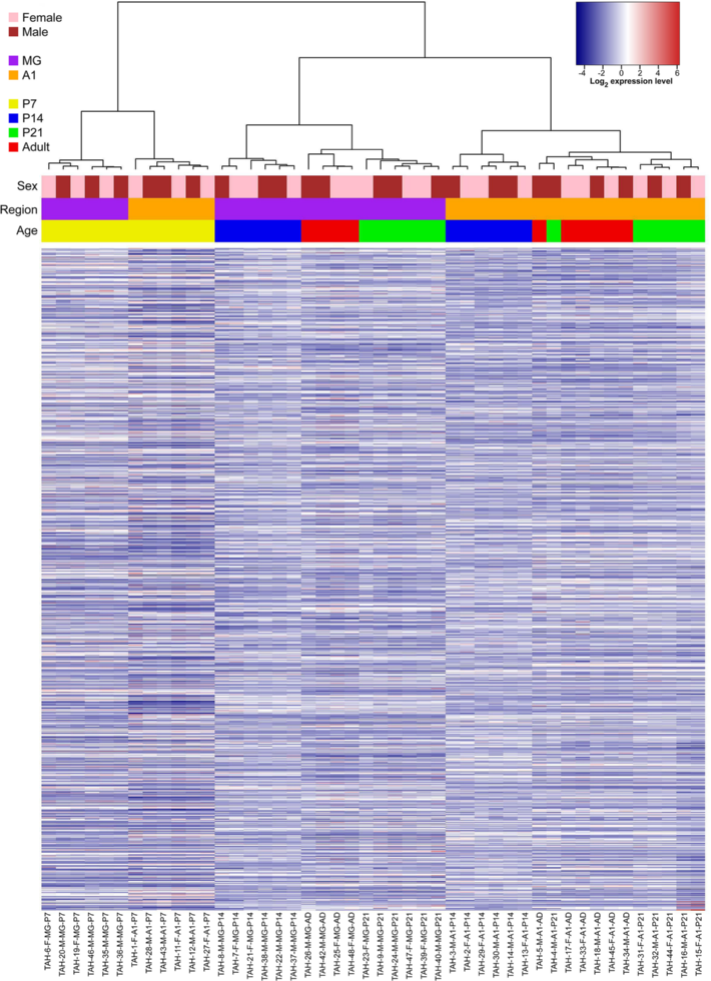
Grand summary of global gene expression in MG and A1 from P7 to adult. (*Top*) Unsupervised hierarchical clustering of samples by sex, brain region, and age. (*Bottom*) Heatmap summarizing total gene expression for each sample, arranged in columns by cluster. Each bar represents one gene. Color code denotes expression level

The RNAseq data went through multiple stages of thorough quality control as recommended by Guo et al. [39]. Raw data and alignment quality control were performed using QC3 [40], and gene quantification quality control was conducted using MultiRankSeq [41]. Raw data were aligned with TopHat2 [42] against mouse mm10 reference genome, and read counts per gene were obtained using HTSeq [43]. Default settings were used for MultiRankSeq, TopHat2, and HTSeq. Normalized counts (used in all plots) were obtained by normalizing each gene’s count against the sample’s total read count, then multiplying by a constant (1 X 10^6^). Hierarchical clustering analysis and heatmaps were produced using the Heatmap3 [44] package from R (Fig. 1). For all samples, quality control data are contained in Additional file 2: Tables S2 – S3. The raw counts are contained in Additional file 2: Table S4. Differential expression analyses between all postnatal ages and brain regions were performed using MultiRankSeq [41], which combines three independent methods for RNAseq analysis: DESeq [45]; EdgeR [46]; BaySeq [47]. These three methods were chosen based on results of several previous studies in which multiple RNAseq differential analysis methods were compared for accuracy and sensitivity of read count-based data [48–52]. In analyses of the same dataset, the methods typically differ in numbers of differentially expressed genes identified in a comparison of any two samples, and also in direction of expression (up- or down-regulation). The false discovery rate (*FDR < 0.05*) was used to correct for multiple testing, and a given comparison was considered to be significant if all three methods identified it as significant. The differential expression data associated with each pairwise comparison (4 ages X 2 brain areas) are summarized in the Results section, with complete data for all genes for all comparisons contained in Additional files 3, 4, 5, 6, 7: Tables S5 – S20. These Additional files are Excel workbooks, organized by tabs corresponding to each supplementary Table. Within each of these files, the listing of single genes is ordered from the smallest to highest numerical ranking (i.e., highest to lowest degree of differential expression), based on p-values from DESeq, EdgeR, and BaySeq. The order can be changed with sorting and filtering functions in Excel.

### Validation of sequencing

Validation of sequencing was accomplished by in situ hybridization (ISH) of 4 genes (*Gapdh, Slc32a1, Slc17a6, Slc17a7*) in A1 and MG, also profiled in a related study of their maturational trajectories, regional patterns of expression, and co-expression within single neurons. Full methodological descriptions of the tissue processing, primer sequences, in situ hybridization, and quantification are available in Hackett et al. (2015, in press) [53]. Briefly, 3 animals in each age group from the same breeding colony were euthanized and perfused transcardially with 4 % phosphate buffered paraformalin. The extracted brains were sectioned at 50 μm in the coronal plane. Single colorimetric in situ hybridization was performed on sequential tissue sections processed for each gene (Additional file 8: Figure S2, top). Expression levels were quantified by densitometric measurements in regions of interest confined to A1 and MG. Raw grayscale intensity of the three target genes (*Slc32a1, Slc17a6, Slc17a7)* was background corrected and normalized by *Gapdh* grayscale intensity, which did not change significantly during development in either region by RNAseq or ISH.

These 4 genes are particularly useful for validation as they have distinct patterns of expression in A1 and MG and a documented maturational time-course. The housekeeping gene (*Gapdh*) is widely expressed in all neurons. It had a flat maturational trajectory and was used for normalization of ISH for the other genes. *Slc32a1 (*aka *VGAT)* is expressed at moderate levels in A1 and low levels in the MG. *Slc17a7 (*aka *VGluT1*) is expressed at high levels in cortex and low in the MG, whereas the expression of *Slc17a6 (*aka *VGluT2*) is complementary in these structures. Additional file 8: Figure S2 (bottom) contains plots of expression levels comparing quantification of the sequencing and in situ hybridization. These data indicate good agreement between methods with respect to both regional differences in expression and the maturational trajectories.

### Functional gene set analyses (GSEA)

Functional gene set enrichment analysis (GSEA) is widely used to characterize enrichment of functionally-related sets of genes in a sample [54]. In this study, GSEA was used to rank genesets by enrichment magnitude and indicate whether the geneset was up- or down-regulated. GSEA was conducted on geneset listings drawn from two sources as of September 2014: (1) the Gene Families database maintained by the *HUGO Gene Nomenclature Committee at the European Bioinformatics Institute* (http://www.genenames.org) (HUGO) [55]; and (2) Mouse Genome Informatics (MGI) Gene Ontology Browser, maintained by Jackson Laboratories (www.informatics.jax.org) [56]. The HUGO database organizes the genome by gene family (e.g., ion channels, receptors, zinc finger proteins, etc.). The MGI database organizes genes into functional categories, where each geneset may contain genes from multiple gene families. Both of these databases are constructed and updated by consortium contributors based on review of the primary literature.

GSEA was applied to 111 gene families from the HUGO database and 51 Gene Ontology (GO) categories from the MGI database. Categories were selected for relevance to brain development and structure, synaptic transmission, and synaptic plasticity. Additional file 9: Table S21 contains the normalized counts of all samples for the 19,826 genes currently listed in the entire HUGO database, organized alphabetically by gene name. From this listing, a subset of 1557 genes within 111 gene families related to brain maturation and function were used to generate Additional file 9: Table S22, which contains the complete GSEA results for these gene families, ordered by FDR value. From the MGI database, Additional file 9: Table S23 contains normalized counts for 1402 genes distributed within the 51 GO categories selected (note that some genes are members of more than one GO category). The complete GSEA results are contained in Additional file 9: Table S24.

### Construction of a gene families database

A major goal of this study was to develop a repository of the entire dataset formatted in a manner that simplified the screening and extraction of data at the global and single gene levels. The intent was to enable users to identify regional and age-related expression in genes of interest without extraction and analysis of the raw data (although the raw data are also available for such purposes). One of the most extensive and accessible resources provided is organized by gene family, and is contained within a single file (Additional file 10: Table S25). Approximately 4700 genes within 237 gene families listed in the HUGO database are organized into 20 functional groups, segregated by tabs. For each gene family, the normalized read counts of each member gene are tabulated and plotted as a function of postnatal age and brain region. There are 145 graphs, each plotting normalized read counts in A1 and MG as a function of postnatal age. The unique format of Additional file 10: Table S25 permits rapid inspection of the maturation trends for individual genes and gene families for both brain regions. As a guide to the use of this resource, an index with instructions is included under the first tab entitled, “Read Me + Index”. Two of the gene families contained in Additional file 10: Table S25 are highlighted in the Results section below.

### Look-Up tool for generating maturational profiles at the single gene level

To facilitate screening and extraction of profiles from the database, a Look-Up tool was developed (Additional file 11). The tool automatically plots the maturational profiles and correlation matrices for any single gene or list of genes (up to 25 at a time). It also generates a listing of the normalized counts for all samples for extraction for other purposes.

## Results

### Data quality

RNAseq data were obtained from 48 samples and quality controlled. Samples information (sample ID, brain region, age, sex, and quality assessments) are contained in Additional file 2: Table S1. On average, sequencing produced 33.8 million reads per sample (range: 27.6–45.1 million). Sample 10 failed sequencing with less than half million reads produced, and sample 41 had relatively low read counts. Both were removed from subsequent analyses. No other quality issue was observed. The raw data statistics are contained in Additional file 2: Table S2. Alignment quality control was conducted, revealing that an average of 77.19 % of all reads (range: 51.86–83.01 %) were aligned to coding RNA regions (Additional file 2: Table S3). The complete raw read count information can be found in Additional file 2: Table S4.

### Hierarchical clustering and differential expression analyses

Comparative transcriptomic analyses over all samples indicated that global gene expression patterns varied by region and postnatal age. Unsupervised hierarchical clustering analysis (Fig. 1) revealed several global trends. First, gene expression patterns were dominated by age at P7, but by brain region from P14 to adult. At P7, samples were clustered by age, then by brain region. From P14 to adult, samples were almost perfectly separated into two large clusters by region, and then by age within each regional cluster. Only a single sample (P21, A1) clustered with another age group (Adult, A1). By comparison, samples did not cluster by sex within any brain region or postnatal age. Overall, then, clustering was dominated by brain region and postnatal age.

**Fig. 2:**
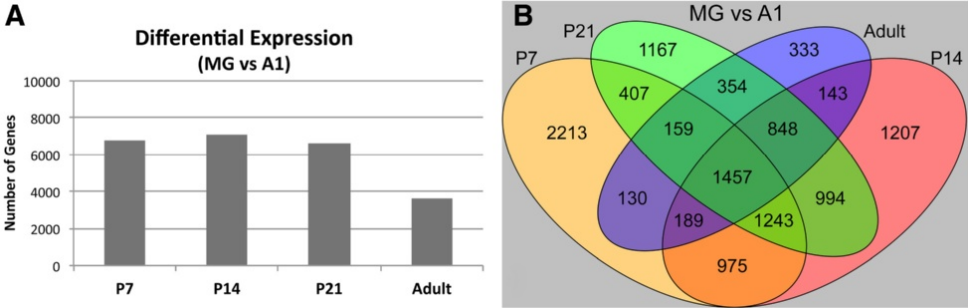
Differential expression in A1 and MG. **a** The total numbers of differentially expressed genes between A1 and MG are plotted for each postnatal age. **b** Overlapping differential expression in A1 and MG. The Venn diagram depicts the total numbers of genes that were differentially expressed (MG vs A1) at only one postnatal age, and the numbers that were commonly expressed in all age group combinations. See text for proportions

**Fig. 3:**
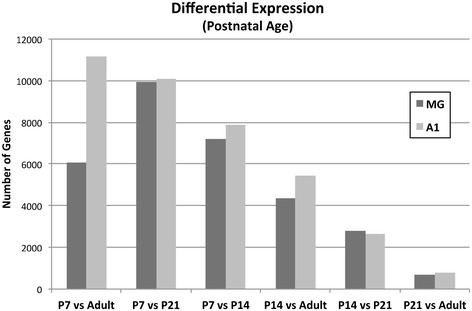
Differential expression between age groups. The total numbers of differentially-expressed genes are plotted for each of the six possible comparisons. Comparisons with P7 yielded the largest numbers of differentially expressed genes, and totals declined with increasing age

To further explore these observations, *differential expression analyses* were systematically conducted comparing brain region and postnatal age (Figs. 2 and 3). Complete results for all comparisons (including fold change and p-values for all genes) are contained in Additional files 3, 4, 5, 6 and 7: Tables S5-S20. These analyses revealed several trends, described in the next two sections.

### Differential expression between brain regions

First, comparing A1 and MG, regional differences in expression were substantial at all ages (Fig. 2a). The total numbers of differentially expressed genes were comparable from P7 to P21 (P7: 6773; P14: 7056; P21: 6629), then dropped to nearly half by adulthood (Adult: 3613). This indicates that regional differences in gene expression are greatest during postnatal development, but remain significant in adulthood. The Venn diagram in Fig. 2b depicts the total numbers of differentially expressed genes that were unique to each age, and those that were also differentially expressed in at least one other. The totals indicate that about 20 % of differentially expressed genes were unique to one age group, while a majority of those identified was common to more than one age group (P7, 67 %; P14, 83 %; P21, 82 %; Adult, 91 %; overall, 80 %). Fewer genes were differentially expressed in more than 2 age groups, however (3 ages, 10 %; all ages, 6 %). Overall, these data reflect robust regional differences in gene expression at all ages, and account for much of the regional clustering observed in Fig. 1.

In Table 1, the top 50 differentially expressed genes between MG and A1 are listed by postnatal age. Ranking was based on the *p-values* returned by DESeq2, EdgeR, and BaySeq analyses. Genes in the first four columns (left) were more highly expressed in the MG, whereas genes in the next four columns (right) were more highly expressed in A1. Several notable trends were observed. First, the genes in each listing (column) represent multiple gene families. Rarely was a single gene family represented more than once. This indicates that regional differences in gene expression were broadly distributed across multiple gene families. Second, about one-third of the genes listed in each column (i.e., age group) were also listed in at least one other (MG > A1, 33 %; A1 > MG, 35 %). Comparing P7 with adult in both regions yielded the least number of duplicated genes (*N* = 8, 16 %), suggesting greater diversity in the most highly expressed genes for that age interval compared with the others. Only two genes were more highly expressed in the MG than A1 at all ages (*Slitrk6, Vav3*). Four genes had higher expression in A1 than MG at all ages (*Met, Efcab6, Hs3st2, Scube1*). These genes are unique in that they ranked among the top differentially expressed genes between regions in the entire transcriptome across the entire age range. Of these, *Slitrk6* and *Met* have been intensively studied and found to be critical for normal development in the forebrain [57–63].

**Table 1:** Top 50 differentially expressed genes in MG and A1

MG > A1				A1 > MG			
P7	P14	P21	Adult	P7	P14	P21	Adult
*Agt*	*Igsf1*	*Tnnt1*	*Tcf7l2*	*Met*	*Met*	*Mpped1*	*Mpped1*
*Zmat4*	*Ntng1*	*Vav3*	*Slc17a6*	*Sla*	*Nov*	*Rasgef1c*	*Sema3a*
*Rgs8*	*Cachd1*	*Igsf1*	*Agt*	*Mef2c*	*Satb2*	*Chrm4*	*Satb2*
*Lhx9*	*Pappa*	*Shisa6*	*Zfhx3*	*Sema3a*	*Mpped1*	*Met*	*E130012a19rik*
*Id4*	*Prkch*	*Amotl1*	*Prox1*	*Gda*	*Mef2c*	*Ngef*	*Nov*
*Aw551984*	*Plekhd1*	*Inadl*	*Tanc1*	*Pde2a*	*Sema3a*	*Pdzrn3*	*Cckbr*
*Zfhx3*	*Zic1*	*Itih3*	*Vav3*	*Satb1*	*Kcnf1*	*Scube1*	*Mef2c*
*Baiap3*	*Cacng5*	*Fhdc1*	*Synpo2*	*Mlip*	*Fam81a*	*Cckbr*	*Ngef*
*Slc6a4*	*Inadl*	*Ret*	*Srgap1*	*Kcnf1*	*Foxp1*	*Foxp1*	*Dkkl1*
*Calb2*	*Prox1*	*Tanc1*	*Clmn*	*Dok5*	*Gm11549*	*Kcnf1*	*Lamp5*
*Slitrk6*	*Synpo2*	*Pcp4l1*	*Cacng5*	*Dnah5*	*Satb1*	*Efcab6*	*Bmp3*
*Rbms3*	*Lef1*	*Nell1*	*Adra2b*	*Baiap2*	*Ipcef1*	*Rasl11b*	*Efcab6*
*Epb4.1l4b*	*Vamp1*	*Rab37*	*Frem3*	*Ipcef1*	*Tiam2*	*Unc5d*	*Atp6ap1l*
*Cachd1*	*Slc17a6*	*Zfp423*	*Zic1*	*Phyhip*	*Vip*	*Atp6ap1l*	*Stx1a*
*Car10*	*Tcf7l2*	*Synpo2*	*Itih3*	*Lmo4*	*Tbr1*	*Hs3st2*	*Arpp19*
*Vav3*	*Calb2*	*Lef1*	*Slitrk6*	*Nrip1*	*Chrdl1*	*Myl4*	*Homer1*
*Frem3*	*Vav3*	*Gdf11*	*Syt9*	*Tiam2*	*Efcab6*	*Pak6*	*Rasgef1c*
*Arhgap24*	*Sema5a*	*Grm4*	*Vangl1*	*Cnksr2*	*Atp1a1*	*Satb1*	*Rasl11b*
*Pappa*	*Frem3*	*Tcf7l2*	*Rora*	*Cd24a*	*Kcnj4*	*E130012a19rik*	*Dtl*
*Epha10*	*Slitrk6*	*Epn3*	*Onecut2*	*Kcnv1*	*Plk2*	*Kcnh3*	*Cdkl4*
*Nell1*	*Tanc1*	*Plekhd1*	*Sparc*	*Efcab6*	*Ngef*	*Efnb2*	*Met*
*Sash1*	*F13a1*	*Trpm3*	*Inpp4b*	*Sstr4*	*Efnb2*	*Stx1a*	*Exph5*
*Rora*	*Pde1c*	*Rbms3*	*Inadl*	*Thrb*	*Cckbr*	*Dtl*	*Col19a1*
*Tshz1*	*Vangl1*	*Cacng5*	*Abhd12b*	*Foxp1*	*Phactr1*	*Olfm2*	*Atp2b4*
*Hap1*	*Amotl1*	*Mcf2*	*Prkch*	*Fbxw7*	*Thrb*	*Fam81a*	*Ipcef1*
*Slc18a2*	*Lhx9*	*Cachd1*	*Grid2ip*	*Gm26937*	*Fmn1*	*Syt16*	*Tbr1*
*B3galt5*	*Nell1*	*Zfhx3*	*Adamts19*	*Jph1*	*Mlip*	*Dclk3*	*Vip*
*Foxp2*	*Slc6a11*	*Nefh*	*Fhdc1*	*Gucy1a3*	*Sla*	*Exph5*	*Cd34*
*Igsf1*	*Tnnt1*	*Trim67*	*Fam19a4*	*Dlgap2*	*Rasl11b*	*Chrdl1*	*Ltk*
*Zfp423*	*Slc6a9*	*Ttyh2*	*Gm16148*	*Hs3st2*	*Atp6ap1l*	*Tmem132d*	*Chrm4*
*Plcb4*	*Ablim3*	*Arhgap24*	*Edaradd*	*Fam49a*	*Syt16*	*Arl4d*	*Arhgap10*
*Cdh4*	*Gpr116*	*Prkch*	*Scube2*	*Ptprk*	*Scube1*	*Doc2a*	*Cabp1*
*Klhl13*	*Shox2*	*Fign*	*Ano5*	*Phactr1*	*Cnksr2*	*Grm3*	*Dact2*
*Cacna2d2*	*Col25a1*	*Cpne7*	*Slc6a11*	*Ndrg1*	*Exph5*	*Ppp1r1b*	*Tmem132d*
*P2ry1*	*Arhgap24*	*Adra2b*	*Ret*	*Kcnj4*	*Kcnh3*	*Nlk*	*Vill*
*Pde1c*	*Syt9*	*Plxdc1*	*Pappa*	*Trpa1*	*Rprml*	*Meis2*	*Meis2*
*Col25a1*	*Hapln4*	*F2r*	*Vash2*	*Scube1*	*Prkcb*	*Arpp19*	*Hkdc1*
*Bcas1*	*Slc29a1*	*Grid2ip*	*Slc29a1*	*Cntn3*	*Camk2n1*	*Kcnh7*	*Tshz3*
*Plxnb3*	*Cpne9*	*Slitrk6*	*P2ry1*	*Sox5*	*Gm872*	*Bmp3*	*Kcnh7*
*Gbx2*	*Plcb4*	*Vash2*	*Mctp2*	*Adamts3*	*Pde2a*	*Dact2*	*Kcnh4*
*Cnp*	*Sgpp2*	*Sgpp2*	*Glra1*	*Ntsr1*	*Kcnt2*	*Lzts1*	*Hs3st2*
*Arhgef16*	*Rimkla*	*Sema5a*	*Dusp27*	*Kcnq5*	*Hs3st2*	*Boc*	*C130074g19rik*
*Pik3r3*	*Rab37*	*Wnt3*	*Adamts15*	*Etv6*	*Fbxw7*	*Pou6f1*	*Scube1*
*Col11a2*	*Rbms3*	*Lhx9*	*Adarb1*	*Vcan*	*Lmo4*	*Tshz3*	*Egr3*
*Vamp1*	*Fhdc1*	*Clmn*	*Ramp3*	*Mapk15*	*Tshz3*	*Osbpl10*	*Camk2n1*
*Irx2*	*Rasa4*	*Srgap1*	*Grm1*	*Nr2f1*	*Tgm3*	*Kcnh4*	*Tgm3*
*Btbd11*	*Ptpn3*	*Rimkla*	*Zfp804a*	*Nudt4*	*Bcl11a*	*Tgm3*	*Lzts1*
*Alk*	*Adamts15*	*Fads6*	*Lef1*	*Rhou*	*Cdk17*	*Arhgap10*	*1110032f04rik*
*Itpr2*	*Gbx2*	*Scube2*	*A2m*	*Vip*	*Ephx4*	*Sowahb*	*Atp1a1*
*Cdh22*	*P2ry1*	*Vipr2*	*Wnt3*	*Plk2*	*Lzts1*	*Tcap*	*Ankrd63*

The top 50 differentially expressed genes between MG and A1 are listed by postnatal age. *Left columns*, genes with significantly higher expression levels in MG compared to A1. *Right columns*, genes with higher expression levels in A1 compared to MG. Significance (*p < 0.05*) and ranking determined by differential expression by DESeq2, EdgeR, and BaySeq. Genes listed in more than one age group are noted in the text. Non-coding and genes of unknown type were excluded

### Differential expression between age groups

To further elucidate the age-related changes in A1 and MG, differential expression analyses were conducted between age groups *within* each region. In Fig. 3 and Table 2 (top panel), the numbers of differentially expressed genes are given for comparisons of each age with all other ages. Several trends were observed. First, comparisons of all other ages with P7 yielded the largest numbers of differentially expressed genes. The fewest differentially expressed genes were found in comparisons between older animals. Similarly, for the comparisons between *successive* age groups (i.e., P7-P14, P14-P21, P21-Adult), the number of differentially expressed genes declined steadily with increasing age in both regions. These trends reflect the stabilization of gene expression levels with increasing age. Second, with one exception (P7-Adult), the numbers of differentially expressed gene in MG and A1 was comparable for each age comparison. This suggests that gene expression matures at a comparable rate in both regions.

**Table 2:** Differential expression by age group and brain region

	A1			MG		
Age	P14	P21	Adult	P14	P21	Adult
P7	7886	10099	11181	7187	9957	6071
P14	---	2648	5417	---	2770	4380
P21	---	---	769	---	---	689
	A1					
Age	P7-P21	P7-Adult	P14-P21	P14-Adult	P21-Adult	
P7-P14	6590	6412	1684	2844	457	
P7-P21	---	8473	2280	3627	515	
P7-Adult	---	---	2214	4230	594	
P14-P21	---	---	---	2247	275	
P14-Adult	---	---	---	---	657	
	MG					
Age	P7-P21	P7-Adult	P14-P21	P14-Adult	P21-Adult	
P7-P14	6203	3940	1667	2241	394	
P7-P21	---	5279	2377	3099	480	
P7-Adult	---	---	1833	2552	387	
P14-P21	---	---	---	2136	292	
P14-Adult	---	---	---	---	588	

In each panel, the number of differentially expressed genes are given for each comparison within the brain region indicated (A1 or MG). *Top*, comparisons of each age with all other ages. These totals are plotted in Fig. 3. *Middle (A1)* and *bottom (MG)*, interactions between all possible age group comparisons. Totals reflect the numbers of differentially expressed genes from each age group comparison (e.g., P7-P14) that were also differentially expressed in all others (e.g., P7-P21, P7-Adult, etc.)

In the middle and bottom panels of Table 2, the totals reflect the number of genes that were differentially expressed at more than one age interval. That is, how many of the differentially expressed genes identified from comparisons between each age group (e.g., Fig. 3) were also differentially expressed at the other age intervals? Trends in these data are less obvious, but two observations are worth noting. First, substantial numbers of the same genes are differentially expressed in more than one age interval (A1 range: 275–8473; MG range: 292–6203). Secondly, these totals were lowest for comparisons involving the older age groups (e.g., P21, adult), and declined with age in a manner that was proportional to the numbers of differentially expressed genes compared. This is most clearly visualized from interactions with P21-Adult (last column), where totals in both brain regions reach minimum values. Overall, these findings support the general conclusion that changes in gene expression decline with age in both regions.

**Fig. 4:**
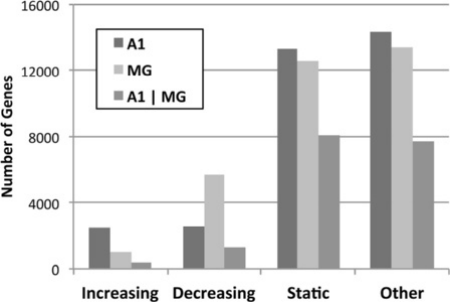
Expression trend analysis. The number of genes with increasing, decreasing, static or other maturational trajectories is plotted for A1 and MG. Also plotted are the numbers of genes with these profiles in both A1 and MG

In Tables 3 and 4, the top 50 up-regulated and down-regulated genes in A1 and MG are listed for each of 4 maturation intervals (P7-Adult, P7-P14, P14-P21, P21-Adult). Rankings were based on the *p-values* from the 3 differential expression analysis methods (see methods). Although many hundreds of genes had increasing or decreasing trajectories (see Fig. 4), these truncated listings are instructive in that they reflect patterns in the regional and maturational changes observed.

**Table 3:** Top 50 up-regulated genes between age groups in A1 and MG

A1				MG			
P7 vs Adult	P7 vs P14	P14 vs P21	P21 vs Adult	P7 vs Adult	P7 vs P14	P14 vs P21	P21 vs Adult
*Ankrd33b*	*Ankrd33b*	*Lzts3*	*Rims3*	*Trf*	*Cnp*	*Mal*	*Qdpr*
*Lamp5*	*Fam107a*	*Hapln4*	*Crebrf*	*Grin2c*	*Mobp*	*Aspa*	*Crebrf*
*Zmat4*	*Gpr158*	*Bc030499*	*Hsf4*	*Tmem88b*	*Mag*	*Opalin*	*Il33*
*Cap2*	*Sept8*	*Dusp1*	*Cbx7*	*Etnppl*	*Ugt8a*	*Plekhb1*	*Fbxw15*
*Vamp1*	*Extl1*	*Ankrd33b*	*Itpr1*	*Erbb3*	*Mal*	*Ndrg1*	*Serpinb1a*
*Zfp365*	*Cap2*	*Hipk4*	*Aifm3*	*Galnt6*	*Nfasc*	*Mog*	*Upp2*
*Cpeb1*	*Ypel2*	*Etnppl*	*Tle6*	*Hapln2*	*Mog*	*Synj2*	*Pex5l*
*Extl1*	*Lamp5*	*Mei1*	*Ankrd12*	*Itih3*	*Ermn*	*Cpm*	*Cbx7*
*Rasgrf1*	*Tmem132d*	*Igfn1*	*Crebl2*	*Plekhb1*	*Fam107a*	*Abhd12b*	*Pkd2l1*
*Lrrk2*	*Car10*	*Cpne9*	*Lamc2*	*Fam107a*	*Sept4*	*Tmem63a*	*Car2*
*Faim2*	*Lynx1*	*E130012a19rik*	*Hapln4*	*Abhd12b*	*Tppp*	*Plp1*	*Abca8a*
*Fam107a*	*Zfp365*	*Bhlhe40*	*Phf15*	*Sec14l5*	*Kcna1*	*Ermn*	*Kif13b*
*Phf15*	*Rasgrf1*	*Aifm3*	*Epdr1*	*Tmem63a*	*Gm15440*	*Cryab*	*Rassf2*
*Gprc5b*	*Scn2b*	*Lamc2*	*Acvr1c*	*Gjc2*	*Scn4b*	*Adamts2*	*Pls1*
*Ypel2*	*Got1*	*Efhd2*	*Aktip*	*Cryab*	*Slc44a1*	*Gpd1*	*Xdh*
*Gpr158*	*Gabrd*	*Ptgs2*	*Resp18*	*Hhatl*	*Tmem88b*	*Adra1b*	*Ankrd12*
*Itpr1*	*P2ry12*	*Tyro3*	*Tle2*	*Plekhh1*	*Acot11*	*Itih3*	*R3hcc1l*
*Sept8*	*Camk2n1*	*Spag5*	*H2-T22*	*Gjb1*	*Gsn*	*Anln*	*Tcf20*
*Egr1*	*Ntsr2*	*Gstm1*	*Upp2*	*Opalin*	*Kcna2*	*Cbx7*	*Phf15*
*Cbx7*	*Grin2a*	*Arhgef25*	*6330403k07rik*	*Acvr1c*	*Opalin*	*Rreb1*	*Pcolce2*
*Camk2n1*	*Camk2a*	*Igfbp6*	*Flywch1*	*Hrh3*	*Gjc2*	*Lpar1*	*Chdh*
*Lzts3*	*Hspa2*	*Phf15*	*Phf1*	*Pvalb*	*Cpox*	*Dock10*	*Cma1*
*Tacc1*	*Tmem38a*	*Ppp1r1b*	*Rpe65*	*Rgs16*	*Ell2*	*Tmem88b*	*Ppp1r3c*
*Car10*	*Lgi3*	*Zfp831*	*R3hcc1l*	*Hlf*	*Slc6a17*	*Faim2*	*Sec11c*
*Kcnab2*	*Pde8b*	*Endou*	*Stard10*	*Kcna1*	*Erbb3*	*Aldh1a1*	*Fmn1*
*Dusp14*	*Kcna1*	*Tnnc1*	*Pms1*	*Gpr37*	*Fa2h*	*Il1rap*	*Atp1b3*
*Scn2b*	*Fam212b*	*Scn1b*	*Xdh*	*Fa2h*	*Secisbp2l*	*Gprc5b*	*Cep112*
*Gpd1*	*Rgs8*	*Lamp5*	*Marf1*	*Napepld*	*Cnnm1*	*Sept4*	*Fth1*
*Unc80*	*Kcnab2*	*Clu*	*Ankrd45*	*Ramp3*	*Ptpn3*	*Ppp1r1b*	*Arl4d*
*Clu*	*Exph5*	*Rims3*	*Tcf20*	*Gprc5b*	*Rnf122*	*Hlf*	*Slc25a13*
*Ano3*	*Mtfp1*	*Scn7a*	*Qdpr*	*Pcp4l1*	*Plekhb1*	*Zfp365*	*Rorc*
*Itm2c*	*Dusp14*	*Gpd1*	*Tnk2*	*Mog*	*Cntnap1*	*Marf1*	*Syt4*
*Marf1*	*Rin1*	*Klhdc7a*	*Stk39*	*Tnnt1*	*Plxnb3*	*Gpr37*	*Mcpt4*
*Flywch1*	*Sstr3*	*Stard8*	*Ntn5*	*Tmem125*	*Lynx1*	*Napepld*	*Oxsr1*
*Rasgef1a*	*Tacc1*	*Ypel4*	*Kif13b*	*Hapln4*	*Cldn11*	*Evi2a*	*Grm3*
*Mbnl2*	*Aldoc*	*Sh2d5*	*Cdyl*	*Fmn1*	*Bhlhe40*	*6330403a02rik*	*Itpr1*
*Pdp1*	*Itm2c*	*Evc2*	*Gstt1*	*Pex5l*	*Plcb4*	*Bc030499*	*Tasp1*
*Crebl2*	*Mbnl2*	*Myh7*	*Bok*	*Bhlhe40*	*Kcnab2*	*Galnt6*	*Pmp22*
*Scn1b*	*Mertk*	*Clec18a*	*Sntb2*	*Kcnj10*	*Slc45a3*	*Tspan2*	*Gstm1*
*Grin2a*	*S100b*	*Dbp*	*Bbx*	*Aspa*	*Sirt2*	*Slit3*	*Mettl7a1*
*Impact*	*Omg*	*Itpr1*	*Cpeb1*	*Tanc1*	*Kcnj10*	*Bhlhe40*	*Bc035947*
*Hipk4*	*Chn1*	*Cbx7*	*Tomm34*	*Plekhg1*	*Zfp365*	*Fam107a*	*Plekhb1*
*Syne1*	*Mag*	*Glt8d2*	*Trim66*	*Vipr2*	*Gjc3*	*Crebrf*	*Ptgds*
*Chn1*	*Atp1a1*	*Rgs14*	*N4bp1*	*Qdpr*	*Eno2*	*Mrvi1*	*Kat6b*
*Rin1*	*Zmat4*	*Csrnp1*	*Stac2*	*Arsg*	*Il1rap*	*Hapln2*	*Ankef1*
*Efhd2*	*Parm1*	*Hba-A2*	*Camkk1*	*Rasd1*	*Mbnl2*	*Gm21984*	*Hsf4*
*S100b*	*Syne1*	*Faim2*	*Ass1*	*Csrp1*	*Rasgrp1*	*Serinc5*	*Pip4k2a*
*Crebrf*	*Necab3*	*Junb*	*Git1*	*Anln*	*Pygm*	*Ypel3*	*Gatm*
*Kcna1*	*Tppp*	*Plxdc1*	*Vamp1*	*Klf9*	*Tmem125*	*Spock3*	*Zfp644*
*Bhlhe40*	*Mbp*	*Pvalb*	*Eif5a2*	*Mrvi1*	*Cldn12*	*Tmod1*	*Zfp109*

The top 50 up-regulated genes in A1 (*left columns)* and MG (*right columns*) were ranked based on differential expression analyses between four successive postnatal age groups (P7 vs Adult, P7 vs P14, P14 vs P21, P21 vs Adult). Significance (*p < 0.05*) and ranking determined by differential expression by DESeq2, EdgeR, and BaySeq. Genes listed in more than one age group are noted in the text. Non-coding and genes of unknown type were excluded

**Table 4:** Top 50 down-regulated genes between age groups in A1 and MG

A1				MG			
P7 vs Adult	P7 vs P14	P14 vs P21	P21 vs Adult	P7 vs Adult	P7 vs P14	P14 vs P21	P21 vs Adult
*Rac3*	*Mex3a*	*Cd24a*	*Marcksl1*	*Rac3*	*Cxadr*	*Lsm11*	*Gpr17*
*Fabp7*	*Casp3*	*Dpysl3*	*Dnmt3a*	*Casp3*	*Tubb2b*	*Nrep*	*Marcksl1*
*Dpysl3*	*Draxin*	*Lsm11*	*Mkrn3*	*Pafah1b3*	*Mapt*	*Kif21b*	*Ugt8a*
*Tnc*	*Cd24a*	*Cxadr*	*Eln*	*Zfp57*	*Dpysl3*	*Ablim3*	*Dnmt3a*
*Dpysl5*	*Trim67*	*Ybx1*	*Col3a1*	*Mkrn3*	*Sbk1*	*Slc6a4*	*Casr*
*Casp3*	*Ndrg1*	*Stmn2*	*Sparc*	*Slc6a4*	*Pafah1b3*	*Dhrs7c*	*Kif19a*
*Trim67*	*St8sia2*	*4930506m07rik*	*9930013l23rik*	*Dpysl5*	*Casp3*	*Bdh1*	*Sirt2*
*Tubb2b*	*Sox11*	*Sla*	*Npnt*	*Panx1*	*Mcm6*	*Gjd2*	*Rassf10*
*Cd24a*	*Vash2*	*Nrep*	*Apc*	*B3gnt5*	*Gpr161*	*Ybx1*	*Itpr2*
*Mtss1*	*Srgap1*	*Tnc*	*Traf3*	*St8sia2*	*Dzip1*	*Inpp5f*	*Rnf122*
*Nrep*	*B3gnt5*	*Gng4*	*Hapln1*	*Myo16*	*Ddah2*	*Dpysl3*	*Ncan*
*Cxadr*	*2410066e13rik*	*Tmsb10*	*Fzd10*	*Top2a*	*Pkia*	*F13a1*	*Tmem141*
*Marcksl1*	*Tes*	*Tet1*	*Tmem229b*	*Gjd2*	*Ybx1*	*Tcerg1l*	*Bdh1*
*Ybx1*	*Dpysl3*	*Erc1*	*Apcdd1*	*Gng4*	*Panx1*	*Stmn2*	*Hmgcs1*
*Panx1*	*Zfp57*	*Tubb2b*	*Aldh1a1*	*Nrep*	*Lin7c*	*Lrrc55*	*Cldn11*
*Vcan*	*Mtss1*	*Tmem229b*	*Gpr17*	*Mex3a*	*Slc6a4*	*Tubb5*	*Fyn*
*Csrp2*	*Sla*	*Met*	*Col5a2*	*Cxadr*	*Armcx6*	*Rac3*	*Nfasc*
*Crmp1*	*Cpne2*	*Tubb2a*	*Emid1*	*Prrt4*	*Stmn1*	*Amer1*	*Gnb4*
*Rimklb*	*Atat1*	*Bdh1*	*Ly75*	*Crabp2*	*Hcn3*	*Ednrb*	*Tmem229b*
*Zfp57*	*Klf8*	*Aplnr*	*Aplnr*	*Ybx1*	*Rimklb*	*Gng4*	*Sema4d*
*Arrdc4*	*Idh1*	*Dpysl5*	*Tet1*	*Aplnr*	*Zfp57*	*Cxadr*	*Enpp6*
*Dok4*	*Csrp2*	*Afap1*	*Bdh1*	*Marcksl1*	*Aw551984*	*Slc1a6*	*Tmem2*
*Hn1l*	*Bzw2*	*Nav1*	*Nav3*	*Tcerg1l*	*Rcn1*	*Slc35f1*	*Col9a3*
*Sema4g*	*Slco5a1*	*Fabp7*	*Cd24a*	*Stk32b*	*Fabp7*	*Zdhhc2*	*5430435g22rik*
*Hmgb3*	*Dcx*	*Dnmt3a*	*Lsm11*	*Sox4*	*Fchsd1*	*Gsg1l*	*Dhcr24*
*Plxna3*	*Gpc2*	*Kif21b*	*Zfp282*	*Tspan6*	*E130309f12rik*	*Col26a1*	*Gsn*
*Idh1*	*Clmp*	*Tuba1a*	*Mfsd2a*	*Rasgef1c*	*Grm3*	*Vgf*	*Gm15440*
*E130309f12rik*	*Hn1*	*Tubb5*	*Nrep*	*Aw551984*	*Prmt2*	*Dpysl5*	*Tspan2*
*C530008m17rik*	*Amer1*	*Rac3*	*Cxadr*	*Hn1l*	*Clmp*	*Cdh13*	*Mog*
*Erc1*	*Tnc*	*Tspan6*	*Kdr*	*Sbk1*	*Idh1*	*Tubb2a*	*Plekha1*
*Flna*	*Epb4.1*	*9930013l23rik*	*Col4a1*	*Fxyd6*	*Plxna3*	*Tmsb10*	*Plp1*
*Cpne2*	*Nrep*	*Trpa1*	*Nid1*	*Dpysl3*	*H2afy2*	*Tet1*	*Man1a*
*Traf4*	*Mapt*	*Dok4*	*Trpa1*	*Rimklb*	*Slc39a6*	*Fstl5*	*Slc45a3*
*Tubb3*	*Fabp7*	*Tubb3*	*Nav1*	*Ablim3*	*Smyd5*	*Col6a3*	*Tns3*
*Dcx*	*Rimklb*	*Af529169*	*Casr*	*Hn1*	*Prune*	*Acat1*	*Idh1*
*Tubb5*	*Panx1*	*Inpp5f*	*Cd93*	*Dzip1*	*Prrt4*	*Mest*	*Slc35b2*
*Sybu*	*Pafah1b3*	*Arhgdig*	*Hkdc1*	*Aspm*	*Mex3a*	*Basp1*	*Rras2*
*Slco5a1*	*Aldh1l2*	*Crmp1*	*Deaf1*	*Rab3b*	*Bzw2*	*Pcdh11x*	*Sh3gl3*
*Tuba1a*	*Bcl7c*	*Acat1*	*Clec2l*	*Vat1*	*4930506m07rik*	*Lin28a*	*Klk1*
*Fam124a*	*Ddah2*	*Ppp1r14c*	*Col9a3*	*Rps6ka6*	*Id4*	*Tubb3*	*Cnp*
*Ddah2*	*Fdps*	*Slc29a4*	*Neu4*	*Cdkn1a*	*Rac3*	*2310003h01rik*	*Barx2*
*Hn1*	*Thbs3*	*Rp23-442i7.1*	*Dio2*	*Prkar2b*	*Maged2*	*Ttc9b*	*Cyp51*
*Vash2*	*Gmip*	*Mkrn3*	*Tmem169*	*Tmsb10*	*Hn1l*	*Pappa*	*Arpc1b*
*Nkain3*	*Znrf2*	*Fam126a*	*Dpysl4*	*Casp7*	*Nln*	*Tubb2b*	*Rap2a*
*Bzw2*	*E130309f12rik*	*Srgap2*	*Slc31a1*	*Olfm2*	*Ctc1*	*Fgd2*	*Hmgcr*
*2410066e13rik*	*Maged2*	*E130309f12rik*	*Elovl2*	*Dcx*	*Gm26512*	*Gm16565*	*Srd5a1*
*Zfp41*	*Impact*	*Aw551984*	*Sema4g*	*Dpysl4*	*Sdc3*	*Cdkn1a*	*Il23a*
*Mex3a*	*Fam124a*	*Tbc1d16*	*Spta1*	*Glra2*	*Rasgef1c*	*Ache*	*Mroh3*
*Gpc2*	*Mt1*	*Grin3a*	*Nov*	*Hcn3*	*Slc1a4*	*Rasgrp2*	*Neu4*
*Stmn2*	*Gabra1*	*Elovl2*	*Gja5*	*Dynlt1-Ps1*	*P2rx2*	*Fndc1*	*Mfsd2a*

The top 50 down-regulated genes in A1 (*left columns)* and MG (*right columns*) were ranked based on differential expression analyses between four successive postnatal age groups (P7 vs Adult, P7 vs P14, P14 vs P21, P21 vs Adult). Significance (*p < 0.05*) and ranking determined by differential expression by DESeq2, EdgeR, and BaySeq. Genes listed in more than one age group are noted in the text. Non-coding and genes of unknown type were excluded

First, note that the genes in each listing represent multiple gene families. Rarely was any single gene family represented more than once for a single comparison. Second, in all maturation intervals, a minority of genes was up- or down-regulated in both A1 and MG (range: 5 to 19). This matches global trends in Fig. 4 and is a further indication of regional specificity in expression trends. Third, relatively few genes were up- or down-regulated in more than one maturational interval (range: 1 to 12). This indicates specificity between age intervals in genes that are differentially expressed. As an example, only 1 gene (*Plekhb1*) was in the top 50 up-regulated genes of all 3 consecutive age intervals (MG, but not A1). Only 2 genes were down-regulated in A1 in all 3 consecutive age intervals (*Cd24a, Nrep*).

### Expression trend analyses

**Fig. 5:**
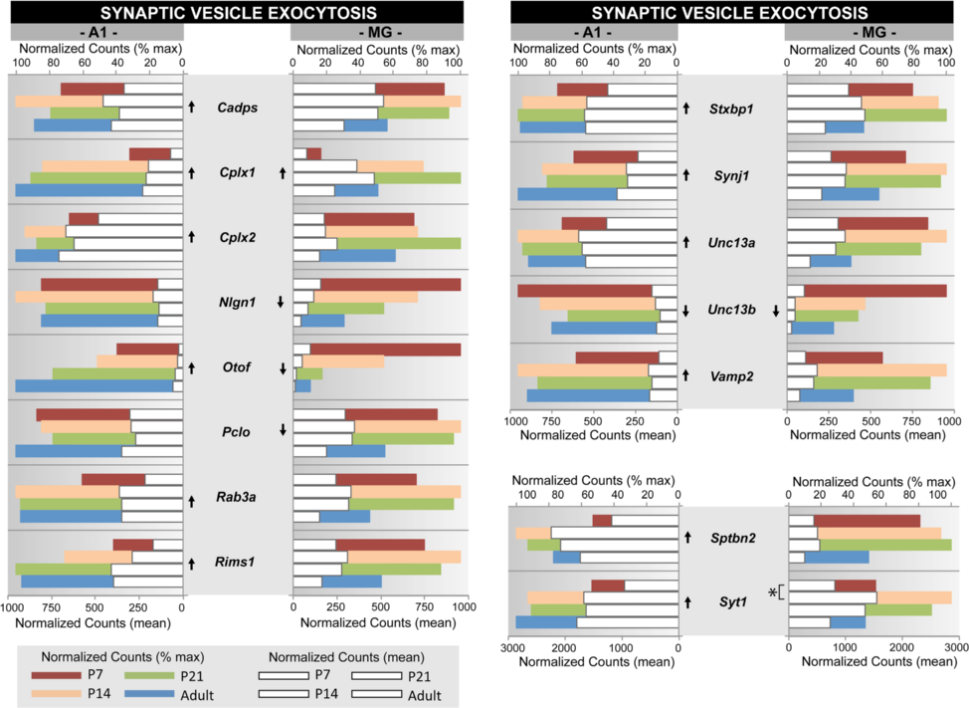
Gene expression profiles of the synaptic vesicle exocytosis gene ontology category. Gene expression profiles are plotted for a subset of genes from one gene ontology category, selected from the GSEA analysis in Table 7 (GO: 0016079, *synaptic vesicle exocytosis*). For each gene, mean normalized counts and % of maximum counts are plotted by postnatal age (P7, P14, P21, Adult) and brain region (A1, MG). Expression trajectory is indicated by arrows (up, down, none). Arrows were included only when differential expression from P7-Adult was significant (*p < 0.05*) by all three methods (DEseq2, EdgeR, and Bayseq)

**Fig. 6:**
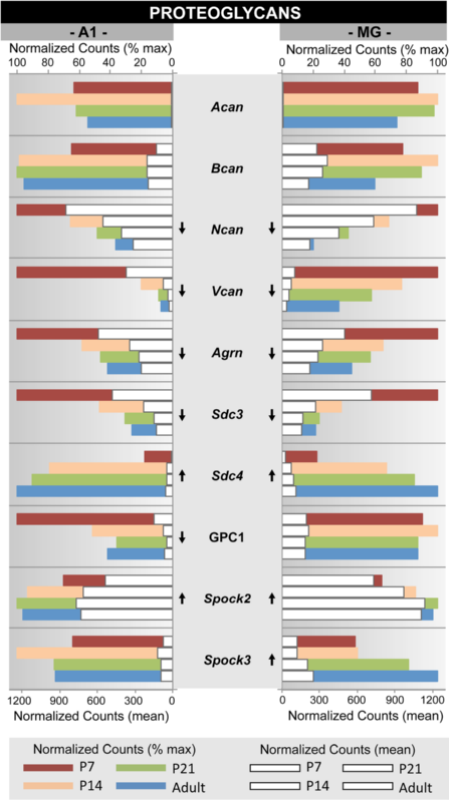
Gene expression profiles of the extracellular matrix proteoglycan family. Gene expression profiles are plotted for a subset of genes from the proteoglycan gene family, which contribute to formation of the extracelluar matrix. For each gene, mean normalized counts and % of maximum counts are plotted by postnatal age (P7, P14, P21, Adult) and brain region (A1, MG). Expression trajectory is indicated by arrows (up, down, none). Arrows were included only when differential expression from P7-Adult was significant (*p < 0.05*) by all three methods (DEseq2, EdgeR, and Bayseq)

**Fig. 7:**
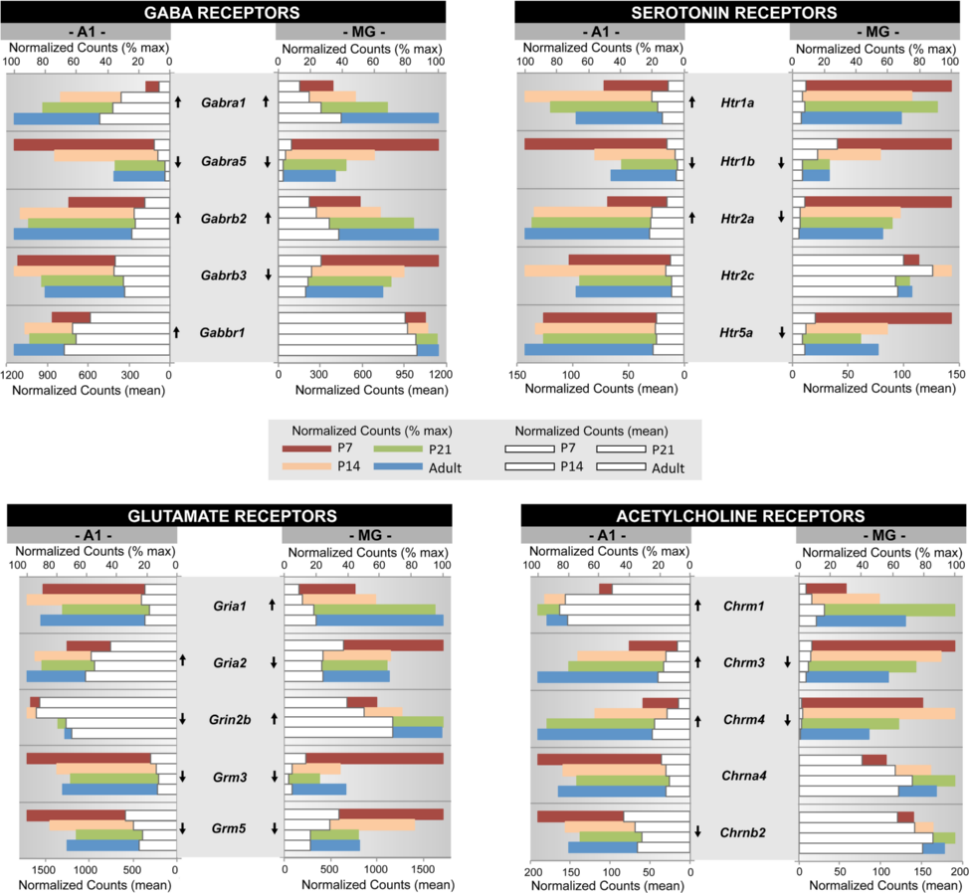
Gene expression profiles of 4 neurotransmitter receptor families. Gene expression profiles are plotted for selected genes from 4 receptor families with roles in neurotransmission and neuromodulation (glutamate, GABA, acetylcholine, serotonin). For each gene, mean normalized counts and % of maximum counts are plotted by postnatal age (P7, P14, P21, Adult) and brain region (A1, MG). Expression trajectory is indicated by arrows (up, down, none). Arrows were included only when differential expression from P7-Adult was significant (*p < 0.05*) by all three methods (DEseq2, EdgeR, and Bayseq)

**Fig. 8:**
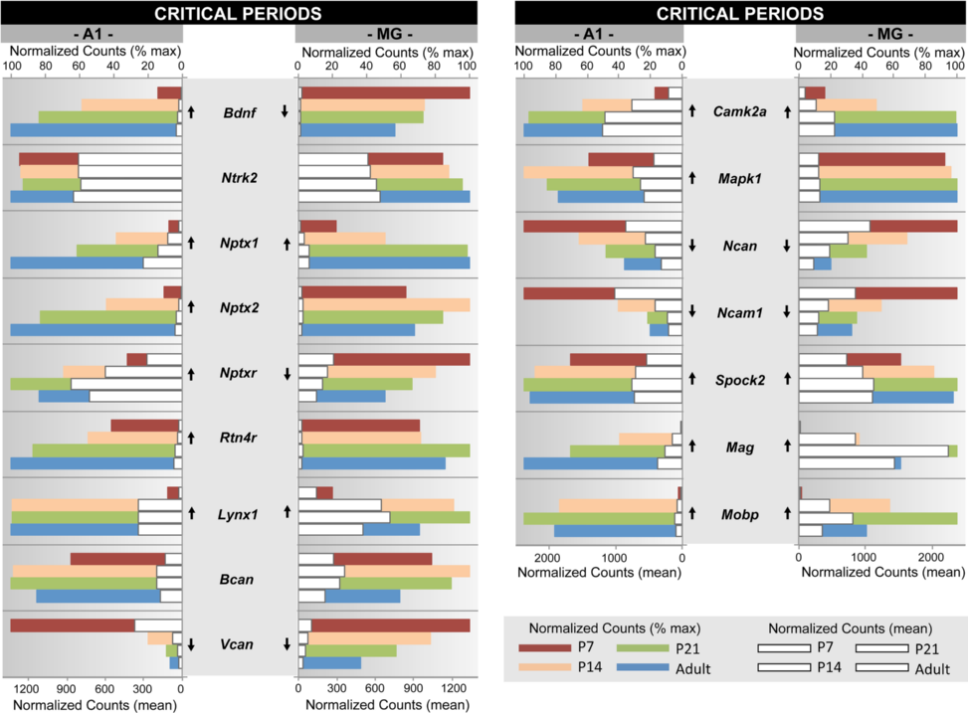
Gene expression profiles of a custom gene ontology category. A subset of genes with established roles in critical period formation in the visual cortex [64, 15] is profiled for A1 and MG. The listing spans multiple gene families. For each gene, mean normalized counts and % of maximum counts are plotted by postnatal age (P7, P14, P21, Adult) and brain region (A1, MG). Expression trajectory is indicated by arrows (up, down, none). Arrows were included only when differential expression from P7-Adult was significant (*p < 0.05*) by all three methods (DEseq2, EdgeR, and Bayseq)

Inspection of expression levels by postnatal age at the single gene level revealed that their maturational trajectories from P7 to adulthood had different profiles. To capture the main patterns, *expression trend analyses* were carried out to identify and tally genes with four different profiles types: monotonically increasing or decreasing, static, and other (Fig. 4, Table 5). A profile was monotonically “increasing” if expression increased successively at each time point and the change between P7 and adult was statistically significant. Monotonically “decreasing” genes were defined in the same fashion, but with decreased expression at each time point. Genes with flat trajectories across all ages were defined as “static”, and those with other patterns of expression (e.g., increasing, then decreasing or decreasing, then increasing) were categorized as “other”. The total numbers of genes with monotonically increasing or decreasing profiles was comparable in A1 (15.3 %) and MG (20.4 %). Of these, nearly equal numbers of genes had increasing and decreasing trajectories in A1, whereas 85.2 % of genes in MG had decreasing trajectories. In comparison to the monotonically changing profile types, the numbers of genes with “static” or “other” profiles were much greater, and similar in both regions. As will be noted in Figs. 5, 6, 7 and 8, a frequently observed profile in the “other” category was characterized by upregulation between P7 and P14 or P14 and P21, followed by downregulation at a subsequent age. Finally, in the third data series (A1 | MG), the number of genes that were differentially expressed in both A1 and MG (i.e., common to both regions) was given for each profile type. These numbers were a variable fraction (between 15 % and 64 %) of the total numbers in either region, depending on the profile. A possible interpretation is that expression of genes with the same maturational trajectory in both regions may be governed by similar factors.

**Table 5:** Expression trend analyses

Trajectory	A1	MG	A1|MG
Increasing	2472	991	382
Decreasing	2546	5691	1286
Static	13372	12586	8092
Other	14310	13432	7709
TOTAL	32700	32700	17469

The total numbers of genes with maturational trajectories categorized as monotonically increasing, monotonically decreasing, static, or other (from P7 to adult) are tallied for A1 and MG. The number of genes that were differentially expressed in both A1 and MG is tallied in the third column (A1|MG). These data are plotted in Fig. 4

### Functional gene set analyses (GSEA)

Functional Gene Set Analysis (GSEA) were performed on 111 HUGO gene families (1557 genes) and 51 MGI gene ontology categories. The data from both resources are described separately below.

### GSEA of MGI gene ontology categories

The complete MGI database contains scores of gene ontology (GO) categories arranged *a priori* by function, rather than by gene family. We performed GSEA on 51 GO categories selected for relevance to brain development, neurotransmission and plasticity (Additional file 9: Table S24). Of these, 20 had a false discovery rate (FDR) of *q < 0.25* (Table 6). The majority of these had upward maturational trajectories, and mainly included genes related to synaptic plasticity and transmission. Among the categories with downward trajectories were those that included genes involved in cell migration, layer formation, axon extension, regionalization, and cell proliferation. The complete listing of genes (including counts) within the MGI GO categories chosen for this study is located in Additional file 9: Table S23.

**Table 6:** GSEA of selected MGI gene ontology categories

Group	Geneset	Size	FDR q-val	Direction
Synaptic plasticity regulation	[GO:0060291] Long-term synaptic potentiation	39	0.0065	UP
Synaptic plasticity regulation	[GO:0048168] Regulation of neuronal synaptic plasticity	50	0.0069	UP
Synaptic plasticity regulation	[GO:0060292] Long term synaptic depression	17	0.0070	UP
Synaptic vesicle function	[GO:0016079] Synaptic vesicle exocytosis	37	0.0075	UP
Synaptic transmission	[GO:0050806] Positive regulation of synaptic transmission	90	0.0093	UP
Synaptic vesicle function	[GO:1902803] Regulation of synaptic vesicle transport	19	0.0241	UP
Synaptic plasticity regulation	[GO:0031914] Negative regulation of synaptic plasticity	4	0.0264	UP
Synaptic transmission	[GO:0001505] Regulation of neurotransmitter levels	135	0.0514	UP
Synaptic transmission	[GO:0050805] Negative regulation of synaptic transmission	41	0.0518	UP
Synaptic transmission	[GO:0035249] Synaptic transmission	77	0.0800	UP
Dendrite development	[GO:0097062] Dendritic spine maintenance	9	0.1191	UP
Synaptic transmission	[GO:0051932] Synaptic transmission	40	0.1696	UP
Synaptic transmission	[GO:0060075] Regulation of resting membrane potential	4	0.1759	UP
Synaptic transmission	[GO:0060078] Regulation of postsynaptic membrane potential [GO:0060079] Regulation of excitatory postsynaptic membrane potential	48	0.1775	UP
Synaptic assembly maturation	Regulation of postsynaptic membrane organization [GO:1901626] and presynaptic membrane organization [GO:1901629]	6	0.2020	UP
Forebrain development	[GO:0021799] cerebral cortex radially oriented cell migration	29	0.1806	DOWN
Forebrain development	[GO:0021794] thalamus development and [GO:0061381] cell migration in diencephalon	11	0.1835	DOWN
Forebrain development	[GO:0021819] layer formation in cerebral cortex	12	0.2085	DOWN
Axon development	[GO:0048675] axon extension	60	0.2292	DOWN
Forebain development	[GO:0021978] telencephalon regionalization	13	0.2375	DOWN

Geneset enrichment analysis (GSEA) of a subset of gene ontology (GO) categories in the Mouse Genome Informatics (MGI) database. From the 51 categories listed in Additional file 9: Table S24, 20 reached the FDR q-value cutoff of 0.25. For each geneset, the number of genes in the group (size), FDR q-value, and direction is given. Categories with upward (UP) and downward (DOWN) maturational trajectories were grouped separately in the Table

### GSEA of HUGO gene families

From the 111 HUGO gene families (1557 genes) analyzed with GSEA (Additional file 9: Table S22), 27 families had a FDR of *q < 0.25* (Table 7). Of these, 5 families had upward maturational trajectories, and contain genes related to neurotransmission or neuronal activity. Of the 22 families with downward trajectories, most are related to intracellular and extracellular structure. Note, however, that Additional file 9: Table S22 contains many gene families that did not reach the 25 % FDR criterion, even though they may contain several genes that were highly expressed, (e.g., all neurotransmitter receptor families other than adrenergic). This typically occurred when multiple members of that family were expressed at low or nominal levels. Similarly, the developmental trajectory of a gene family may not represent all members of that family. Thus, categorization of a family by trajectory or ranking by GSEA may not reflect the profiles of all genes in that family. The complete listing of genes (including counts) within the MGI GO categories chosen for this study is located in Additional file 9: Table S21.

**Table 7:** GSEA of selected HUGO gene family categories

Group	Gene family	Size	FDR q-val	Direction
Ion channels	Potassium channels (KCN)	89	0.005	UP
Receptors (neurotransmission)	Adrenergic Receptors (ADRA, ADRB)	9	0.008	UP
Structural	Lectins, sialic acid binding Ig-like (SIGLEC)	6	0.091	UP
Receptors (peptides)	VIP and PACAP (ADCYAP1) Receptors	3	0.227	UP
Ion channels	Sodium Channels (SCN)	17	0.246	UP
Structural	Collagens (COL)	43	0.000	DOWN
Structural	Kinesins (KIF)	38	0.005	DOWN
Structural (development)	Tubulins (TUBA)	17	0.031	DOWN
Structural	Extracellular matrix proteoglycans	25	0.072	DOWN
Structural	Major cadherins	31	0.075	DOWN
Structural	Protocadherins; non-clustered protocadherins	11	0.090	DOWN
RECEPTORS (peptides)	Vasopressin/oxytocin receptors	4	0.091	DOWN
Structural (development)	Caspases (CASP)	9	0.091	DOWN
Endogenous ligands	ADAM metallopeptidases thrombospondin type 1 (ADAMTS)	19	0.091	DOWN
Structural (development)	Ephrins (EFN)	8	0.094	DOWN
Receptors (other)	Prostanoid receptors	8	0.098	DOWN
Structural	Cadherin-related	16	0.099	DOWN
Structural (development)	EMI Domain Containing (EMID)	7	0.102	DOWN
Receptors (peptides)	Neuropeptide Receptors :	9	0.103	DOWN
Structural (development)	Mex-3 Homologs (MEX3)	4	0.103	DOWN
Receptors (others)	Ephrin Receptors (EPH)	14	0.167	DOWN
Ion channels	Acid-Sensing (Proton-Gated) Ion Channels (ASIC)	4	0.169	DOWN
Ion channels	Chloride Channels (Voltage Sensitive)(CLCN)	8	0.173	DOWN
Receptors (others)	Calcium-Sensing Receptors (CASR)	2	0.186	DOWN
Structural	Cell Surface Proteoglycans	13	0.190	DOWN
Receptors (peptides)	Hypocretin (OREXIN) Receptors (HCRTR)	2	0.195	DOWN
Structural	Dyneins, Axonemal (DN)	14	0.205	DOWN

Geneset enrichment analysis (GSEA) of a subset of gene families in the HUGO database. From 111 categories listed in Additional file 9: Table S22, 27 families reached the FDR q-value cutoff of 0.25. For each gene family, the number of genes in the group (size), FDR q-value, and direction of expression (up, down) are listed. Categories with upward (UP) and downward (DOWN) maturational trajectories were grouped separately in the Table

### Geneset profiling at the single gene level

To fully characterize the data contained within all relevant GO categories and gene families at the single gene level exceeds the scope of any single paper, as many thousands of genes are involved. As an alternative, we created searchable database tables and a look-up tool to permit viewing of the profiles of any single gene or geneset. In addition, the gene families database contains maturational profile plots for about 4700 genes within 237 selected gene families, organized into a searchable database for rapid screening of any gene family (Additional file 10: Table S25).

Examples of these profiles were generated from selected genes within one GO category (GO:0016079, synaptic vesicle exocytosis) and two gene families (extracellular matrix proteoglycans; neurotransmitter receptors) that were found to be enriched by GSEA (Figs. 5, 6 and 7). These three genesets were chosen because they are involved in different aspects of brain maturation (structure and function), and also exemplify the type of information contained within the database. Further, since none of these genesets have previously been profiled in the developing auditory forebrain, the data are both novel and informative. Finally, the profiles selected for illustration are typical of the regional and age-related diversity observed among members of the genesets analyzed in this study, and highlight the importance of evaluating expression patterns individually.

In each of these figures (Figs. 5, 6 and 7), normalized counts (mean and % of maximum) are plotted for each gene by postnatal age and brain region. The mean counts (white bars) convey information about expression magnitude of each gene by age. The % of maximum values (colored bars) facilitate visualization of the maturational trajectories, which are difficult to resolve when genes with high and low expression levels are plotted together. Arrows denote whether expression from P7 to adult was significantly up- or down-regulated.

### Synaptic vesicle exocytosis (GO: 0016079)

Synaptic vesicle exocytosis is the process by which membrane-bound vesicles containing neurotransmitters are directed to their contents at a neuronal synapse. From this GO category (37 genes), the profiles of 15 were plotted in Fig. 5. Overall, this group was up-regulated (Table 6) from P7 to Adult, as might be expected following the onset of auditory experience. At the single gene level, however, profiles were mixed. In A1, 12 of 15 had upward profiles, 2 were static, and only one was significantly down-regulated (*Unc13b*). In MG, only 1 gene was significantly up-regulated (*Cplx1*), 4 were down-regulated, and the remaining 10 were static. The diversity of maturational profiles within this GO category highlights the importance of evaluating trends in expression at the single gene level, as well as by group or family.

One additional comment should be made here before considering other genesets. Note that genes with overall static profiles may exhibit significant changes in expression between one or more age comparisons. For example, the asterisk comparing P7 with P14 for *Syt1* in the MG denotes a significant change for that age interval, whereas comparisons between other age groups were not significant. We did not calculate all 6 of the possible age comparisons for all of the genes profiled in Figs. 5, 6, 7 and 8, but included the *Syt1* example here to draw attention to the potential for such changes at the single gene level of analysis. Also, recall that we used a very strict criterion (the difference must be significant by DESeq2, EdgeR and BaySeq methods), which results in the identification of fewer significant changes.

### Extracellular matrix: proteoglycan family

The proteoglycans are a large class of glycoproteins that contribute to formation of the extracellular tissue matrix surrounding neurons and glia in the brain. Several other gene families are also involved in the formation and maintenance of the extracellular matrix (e.g., collagens, contactins, cadherins, laminins, neural cell adhesion molecules) (see Additional file 10: Table S25). In Fig. 6, 10 proteoglycan genes are profiled. As a family, the proteoglycans were downregulated from P7 to adult, but the profiles of individual genes were diverse. Comparing regions, expression levels and trajectories were fairly symmetric for this set of genes. 5 genes were significantly downregulated in one or both regions (*Ncan, Vcan, Agrn, Sdc3, Gpc1*). Two genes were significantly upregulated in both regions (*Sdc4, Spock2*), and two others had disparate regional profiles (*Gpc1, Spock3*). *Acan* had nominal levels of expression, which was unexpected based on prior studies of visual and somatosensory cortex (see Discussion).

### Receptor families with roles in neurotransmission and neuromodulation

Several major classes of neurotransmitters and neuromodulators are involved in signaling between networks of neurons in the brain (e.g., glutamate, GABA, glycine, acetylcholine, dopamine, serotonin, noradrenaline). Multiple receptor types are associated with each class, forming a large and diverse collection of proteins. None of these families reached the 25 % FDR criterion by GSEA (see Additional file 9: Table S22). Their relatively low ranking in this listing was due to the mixed expression profiles of the genes in these families. Profiled in Fig. 7 are 5 representative genes from each of 4 receptor families with upward (GABA, glutamate) and downward (serotonin, acetylcholine) trajectories by GSEA. The genes were selected to represent the regional and maturational diversity within each family, and for the neurotransmitter receptors, in general. As for the gene families described above, expression levels and maturational trajectories typically varied between genes and brain region. For the GABA and glutamate receptors, expression levels and trajectories were comparable between regions. That is, most of the genes had symmetric expression levels in A1 and MG, and the maturational trajectories of about half were in the same direction (*Gabra1, Gabra4, Gabrb2, Grm3, Grm5*). The remainder had profiles that were opposing (*Gria2, Grin2b*) or mixed (*Gabrb3, Gabbr1, Gria1*). In contrast, there was greater diversity among the serotonin and acetylcholine receptors. For example, expression levels between A1 and MG were asymmetric for most of the genes in these two families. Maturational trajectories were also in opposite directions for three of these genes (*Htr2a, Chrm3, Chrm4*) and non-matching for four others (*Htr1a, Htr5a, Chrm1, Chrnb2*). Overall, these examples illustrate the diversity of expression patterns within each of the receptor families, and reveal that multiple receptor types from several receptor families are expressed in the same brain region. The functional roles of relatively few have been studied in detail, and many are contained within functional GO categories from which their function might be inferred. See Additional file 10: Table S25 to view the profiles of all of the genes within 7 neurotransmitter and 12 neuropeptide receptor families (“Receptors” tabs).

### Custom gene ontology categories

Profiling by gene family is a convenient means of determining which genes within a possibly large family are expressed in the sampled region of interest and how their expression changes with age or some other manipulation. One limitation of this approach is that functionally-related sets of genes belong to multiple families and must be pieced together by additional analysis. By profiling established functional GO categories, genes that are functionally-related (based on prior experimental work) can be explored individually and as a group. Upon closer inspection of the GO categories profiled in Tables 6 and Additional file 9: Table S24, we noticed several trends that could be considered as caveats. First, there is typically some overlap across categories. That is, multiple categories index the same or similar functions, and the same gene or set of genes may be listed in several categories. Second, some GO categories contain a small number of genes (e.g., less than 10), or appear to be constructed from a limited or selective sampling of the literature. Third, the listings are almost always compiled from studies of other brain regions (i.e., non-auditory), some of which have very different patterns of organization (e.g., hippocampus). Thus, for some purposes, it may be advantageous to generate a custom GO category from an established model or a focused literature review.

Profiled in Fig. 8 is a custom GO listing of 16 genes associated with the opening and closing of critical periods plasticity in the visual cortex, based on the model advanced by Takesian and Hensch [64]. Because a comparable model does not exist for auditory cortex, we used this established model to probe our dataset. In this context, *plasticity* refers to the capacity for structural and functional change in some part of the brain (e.g., synapse, circuit, network), as regulated by intrinsic mechanisms or extrinsic factors, such as the onset of sensory experience. *Critical periods* denote periods of time during which the capacity for plasticity is high, and the functional properties of a brain region can be strongly shaped by experience in a manner that has long-lasting or permanent effects [15]. The model described by Takesian and Hensch elucidates the molecular mechanisms associated with the opening and closing of critical periods, which are themselves subject to modification. Because regions of sensory cortex share many features of organization, application of this model to the auditory forebrain may have higher relevance than a GO category based on studies of other brain regions.

As observed for nearly all of the GO categories related to synaptic plasticity (see Table 6), most of the genes had an upward maturational trajectory in one or both regions. 12 of the 16 genes had upward trajectories in A1, but only 5 had upward profiles in both regions (*Nptx1, Lynx1, Camk2a, Mag, Mobp*) (Fig. 8). This appears to reflect regional differences in the genes that govern critical periods. The genes that were up-regulated in A1 are involved in the opening and closing of critical periods in the visual cortex. Nine of these genes were significantly up-regulated between P7 and P14 (*Bdnf, Nptx1, Nptx2, Nptxr, Lynx1, Camk2a, Mapk1, Mag, Mobp*), which correspond to ages before and after hearing onset, and the opening of a critical period for auditory plasticity [13]. One of the genes that was down-regulated in both regions (*Ncam1*, neural cell adhesion molecule) is involved in preventing precocious plasticity prior to the opening of the critical period in visual cortex. The sharp down-regulation of *Ncam1* between P7 and P14 may signal a reduction in its role as an attenuator of plasticity in the auditory forebrain. Conversely, the significant up-regulation between P7 and P14 of two genes related to myelination (*Mag, Mobp*) appears to be related to the ultimate closure of the critical period, as myelin formation dampens plasticity.

**Fig. 9:**
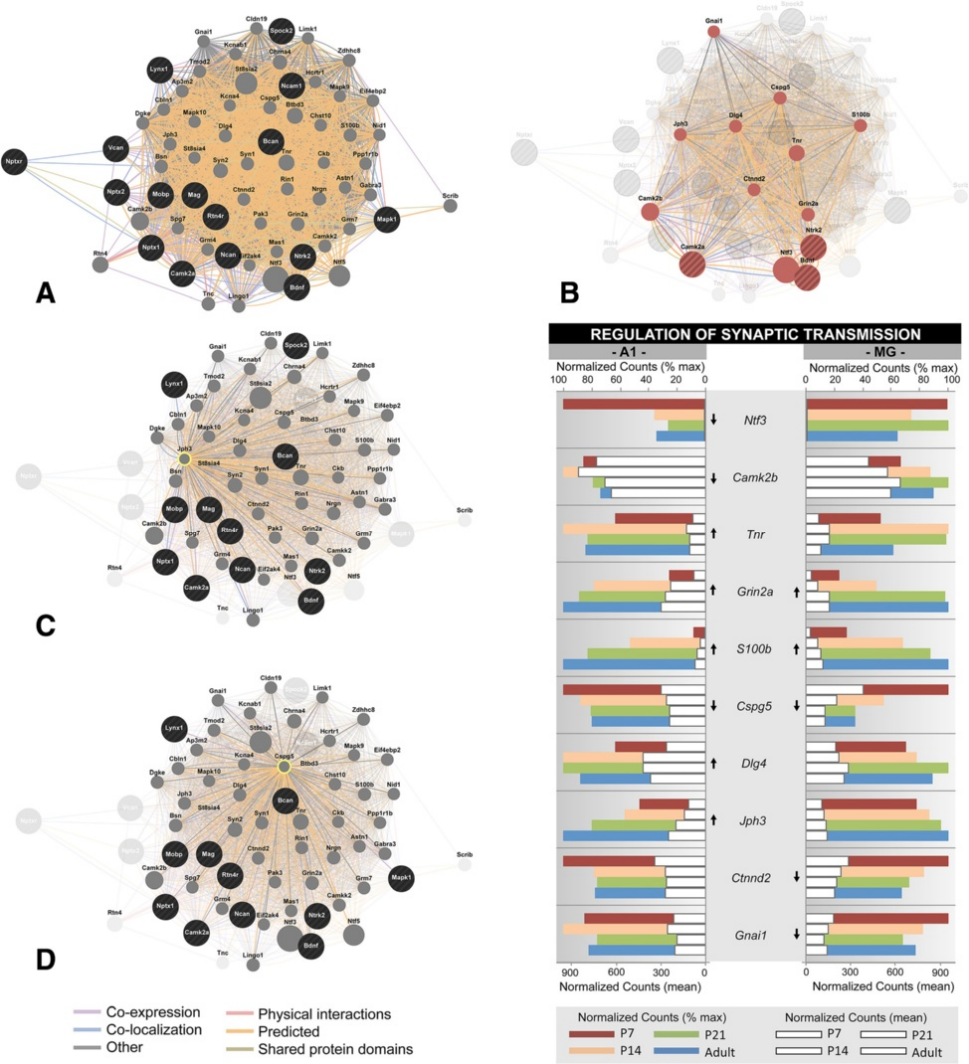
Pathways analysis of critical periods genes. The 16 critical periods genes from Fig. 8 were used as seeds to generate a functional association network of known and predicted interactions using the GeneMANIA tool (http://genemania.org). Analysis based on gene ontology (GO) biological function annotations. **a** Network generated from the 16 critical periods genes (nodes with black circles) and 50 interacting genes (nodes with gray circles). Connection type is denoted by line color (see legend), and strength (line thickness) is weighted by linear regression-based computations of the functional association data in the databases indexed. **b** Connections of the 13 genes (out of 66) from the entire network that were listed in the GO category: *regulation of synaptic transmission*. The normalized counts of these genes are plotted below. **c** *–* **d** Detailed connections of 2 genes from panel **b**: *JPH3* (junctophilin-3)*, CSPG5* (chondroitin sulfate proteoglycan 5). See text for details

To further probe this custom geneset, we used the 16 critical periods genes from Fig. 8 as seeds to generate a functional association network of known and predicted interactions using the GeneMANIA tool (http://genemania.org) [65, 66], based on gene ontology (GO) biological function annotations. The network illustrated in Fig. 9a includes the 16 critical periods genes (nodes with black circles) and 50 interacting genes (nodes with gray circles). The number of interacting genes included is a user-selected option. Connection type is denoted by line color (see legend), and strength (line thickness) is weighted by linear regression-based computations of the functional association data in the databases indexed. In addition to a dense plexus of connections, the 66 genes in this network were cross-listed in 91 GO categories. Figure 9b depicts the connections of 13 genes that were contained within one of these GO categories: *regulation of synaptic transmission*. This includes 3 critical periods genes from Fig. 8 (*Bdnf, Ntrk2, Camk2a*), and 10 interacting genes from the pathways analysis. The normalized counts of these genes are plotted below. Note the expected close association between *Bdnf* (brain-derived neurotrophic factor) and its tyrosine kinase receptor, TrkB (*Ntrk2*).

In Fig. 9c-d, the connections of 2 genes from Fig. 9b are illustrated in detail: *Jph3* (junctophilin-3)*, Cspg5* (chondroitin sulfate proteoglycan 5). Both have significant expression in A1 and MG, and are broadly connected with other nodes in the network. *Jph3* expression increased significantly from P7 to adult in A1 and trended upward in MG. Figure 9c reveals interactions between *Jph3* and 12 of the 13 genes (not *Ntf3*) within the GO category, as well as 56 of 66 genes across the entire network. *Cspg5* expression decreased significantly in both auditory regions. Figure 9d reveals interactions between *Cspg5* and all 13 genes in the GO category, as well as 54 of 66 genes in the network. To date, neither gene has been explicitly linked to critical periods plasticity and functional roles in the auditory forebrain are unknown. Studies of other brain regions, however, indicate that both genes are essential for normal neuronal function. *Jph3* mutations are linked to neuropathological conditions in the brain, such as Huntington’s disease [67, 68]. *Cspg5* plays a role in normal neuronal growth and differentiation [69]. Given these data and their position in the network, further investigation may reveal heretofore unknown roles for one or more of these genes in critical periods plasticity or a related function.

Overall, the analysis illustrated in Fig. 9 provides an example of how transcriptome profiles may be used in conjunction with pathways analysis to guide discovery and generate testable hypotheses. In addition to identification of novel interactions, single gene expression profiles permit identification of genes that are not expressed at significant levels in the brain region of interest. Regionally-specific profiling at the single gene level is essential given the significant differences in expression patterns between regions.

## Discussion

In the present study, we set out to achieve two main goals. The first was to generate complete transcriptome profiles of A1 and MG during postnatal development using next generation sequencing of total RNA. The second was to construct an accessible database in a format that permits extraction and screening of the data for any purpose (Additional files 3, 4, 5, 6, 7, 9, 10 and 11: Tables S5 – S26). Overall, the analyses of global gene expression revealed significant differences between brain regions at all ages, and changes within both regions with postnatal age. Geneset enrichment analyses revealed how those changes manifested within functional categories and gene families. The differential expression and gene families databases permit screening and extraction of the entire dataset down to the single gene level, aided by application of a look-up and plotting tool. To further illustrate the functional relevance and potential applications of the dataset, some of the results are discussed in more detail below.

### Regional differences in gene expression

Regional differences in gene expression are well known in the forebrain [70–72], including numerous genes that have been intensively studied for their roles in brain development [63, 73, 74]. Those roles include both structural (e.g., regionalization, axon guidance, cell differentiation, synapse formation), and functional (e.g., neurotransmission, synaptic plasticity) features, which vary significantly between brain regions. Much progress has been made, but characterization of these features is far from complete for most brain areas.

The present study is the first to profile the transcriptome of the auditory forebrain in any species at any age. In sequencing two anatomically interconnected regions at the same time from the same subjects, we were able to directly compare the magnitude and trajectory of expression in juvenile and mature animals at key time points before and after the onset of hearing. Both hierarchical clustering and differential analyses revealed clear differences in global gene expression between A1 and MG at all ages. While global differences between regions located in the telencephalon and diencephalon are not surprising or especially informative on their own, they reflect important differences in the underlying patterns at the group (gene ontology categories, gene families) and single gene levels. In the present study, regional asymmetries in expression levels and maturational trajectories were commonplace, even for genes of the same family or functional group. Therefore, accurate characterization of the functional roles played by single genes and groups of genes must also account for brain location. This is important, because the regional differences in gene expression are likely to subserve important differences in function. For example, the asymmetrical expression of neurotransmitter receptors in A1 and MG implies that excitatory, inhibitory, and modulatory inputs to each region are mediated by a unique blend of receptor types that variably influence activity [26, 18, 22]. In addition to unique receptor profiles, we also observed regional differences in other genes that directly impact activity (e.g., ion channels, calcium binding proteins). Thus, in addition to differences that are conferred by unique input connectivity, regional differences in function may also be influenced by a rather large set of other factors. Expression profiling provides a way to screen for these factors, and narrow the range of targets for further study.

In addition to differences between the major divisions of the auditory forebrain, we also observed that regional differences in gene expression exist between closely-related structures in the brain. As an example, one of the most widely expressed of the proteoglycans is aggrecan (*Acan*), which has multiple isoforms that are differentially distributed in cortex [75–78]. In studies of visual or somatosensory cortex, *Acan* is upregulated during development and involved in the regulation of critical periods [79, 80]. Matthews et al. (2002) [76] found that *Acan* mRNA levels peak in somato-motor cortex at about P21, where a small subset of cells expressed the gene. Higher expression was noted in subsets of neurons in subcortical motor nuclei and the cochlear nucleus. Ye and Maio (2013) used immunohistochemistry to study development of several perineuronal net components, including *Acan*, in mouse visual cortex from P10 to adulthood. They also found a steady increase in expression that reached a plateau around P28. Based on these studies, we expected to see substantial and increasing levels of *Acan* with age, but this gene was expressed at very low levels in A1 and MG at all ages (Fig. 6). By comparison, the other lecticans (*Bcan, Ncan, Vcan*) were expressed at relatively high levels that changed substantially with age. Turning to the Allen Brain Atlas for additional validation, we observed that *Acan* is expressed in sizable subpopulations of neurons in somatosensory, motor and retrosplenial cortex, moderately in visual cortex, but *rarely* in auditory cortex or MG. Otherwise, the atlas revealed that *Acan* is highly expressed in the thalamic reticular nucleus (TRN) and subpopulations of neurons in major auditory brainstem nuclei (IC, SOC, CN). Thus, *Acan* is expressed at relatively low levels in the auditory forebrain, compared to other central auditory nuclei and sensory cortical areas. More generally, such findings may signal the existence of numerous important differences in structure and function between brain regions – including different sensory systems. While such differences are reminders to use caution when using the findings of one brain region (e.g., visual cortex, hippocampus) to draw conclusions about another (e.g., auditory cortex), the differences offer exciting opportunities for discovery.

### Maturational changes in genes related to neuronal activity and brain structure

Differential analyses of the entire dataset revealed broad-based maturational changes in gene expression in A1 and MG. Globally, samples in both regions were almost perfectly clustered by age group, reflecting distinct patterns of gene expression at each age. The differences between age groups remained robust, despite a steady decline in the total numbers of differentially expressed genes with age.

The greatest changes in global gene expression occurred between P7 and P14, spanning the period before and after eye and ear canal opening in mice. Presumably, some of the changes observed are linked to the onset of sensory experience, especially in gene families related to neurotransmission or synaptic plasticity [81–84]. Also anticipated were changes in genes involved in brain structure, which may be altered as the architecture of each region becomes established. Indeed, the GSEA analyses revealed genes involved in synaptic transmission and plasticity were up-regulated on the whole, while groups of genes involved in establishing brain structure were down-regulated. For example, 14 of the 27 gene families listed in Table 7 contain genes primarily related to structure. All of these families had downward maturational trajectories.

As noted above, however, the expression patterns of individual genes are not always in line with the group as a whole. That is, most groups contained genes with a blend of increasing, decreasing, static and other trajectories. In addition, the distinction between genes with structural and functional roles is not absolute, as multiple functions may be attributed to the products of the same gene. As an example, the proteoglycan gene family (highlighted in the results) is a large and diverse class of glycoproteins known to be major structural components of perineuronal nets (PNNs) and the extracellular matrix in the brain and other tissues [85–88, 76]. The most widely studied are those that carry chondroitin sulfate (CSPG) or heparin sulfate (HSPG) side chains (e.g., hyalectans or lecticans; glypicans; syndecans). In addition to their structural support roles, the proteoglycans have also been intensively studied for their roles in brain development and plasticity [89–93, 80, 94–97]. Note that three of these genes (*Bcan, Vcan, Ncan*) were cross-listed in Fig. 6 (proteoglycans) and Fig. 8 (critical periods). In cortex, PNNs surround specific subsets of neurons, most notably parvalbumin expressing interneurons [77, 98, 99], where they are involved in the transfer of Otx2, which is apparently essential for the opening and closing of critical periods in visual cortex [91, 32]. In the present study, the majority had downward developmental trajectories, consistent with prior evidence that these genes reach peak expression levels at around the time of birth [97, 85, 100, 101]. Exceptions were *Sdc4* and the testicans (e.g., *Spock2, Spock3*), especially *Spock2*, which was expressed at high levels and increased significantly in both regions. Thus, down-regulation of gene expression may signify a decreasing demand for the production of structural proteins as the architecture is laid down, while their persistent expression at relatively modest levels may reflect alternative roles, such as structural maintenance or synaptic plasticity [15, 64].

### Comparison with prior studies of the central auditory pathway

To date, transcriptomic and/or proteomic profiling of the central auditory system remains rather limited. Although no previous studies have focused specifically on the forebrain, it is fortunate that we can at least compare some of our results with studies of auditory brainstem nuclei, where activity-dependent mechanisms were a theme (i.e., postnatal development, hearing loss). These comparisons reveal interesting regional differences in expression within the central auditory pathway, overall, that can improve understanding of the underlying circuits and guide future research.

Kaltwasser et al. (2013) used a proteomic approach to profile over 1200 proteins in the superior olivary complex (SOC) and IC during development (P4 and P60) [34]. The number of differentially regulated proteins between the regions was high (>75 %), whereas less than 20 % had common regulation patterns. Among the upregulated proteins were synaptophysin (SYPH or SYP) and two synaptic vesicle proteins (SV2A, SV2B), which are involved in the regulation of neurotransmission. Our data indicated that all three were greatly upregulated between P7 and P14 in both A1 and MG, and especially in A1 (Additional file 4: Tables S9-S10). The timecourse corresponds to the period of increased synaptogenesis during the early postnatal period [102–104]. In contrast, the cytoskeletal protein Stathmin-1 (STMN1) and calpain-6 (CAN6, CAPN6), involved in cytoskeletal remodeling, were greatly downregulated in the SOC. By comparison, we also observed rapid downregulation of STMN1 between P7 and P14, followed by slower reductions thereafter. Curiously, CAPN6 was only detectable at nominal levels across the entire age range in A1 or MG in the present study, whereas several other calpain genes were strongly expressed in A1 and MG, and exhibited age-related changes in expression (CAPN1, CAPN2, CAPN3, CAPN5, CAPN7*,* CAPN15, CAPNS1) (Additional file 10: Table S25: Calcium Binding & EF Hand tab). As noted earlier, such trends are likely to reflect a fraction of the regional similarities and differences in gene expression that are present.

Ehmann et al. (2013) used microarray analysis to profile gene expression in the rat SOC at P0, P14, P16, and P25. The extensive dataset profiled some 2000 genes, which limits detailed comparisons with the present study within this manuscript [33]. Among the top upregulated genes between P4 and P25 in the SOC were *Mog* and *Mobp*, which are related to myelination. These were also significantly upregulated in A1 and MG in the present study. Several potassium channel genes were significantly upregulated in both studies (*Kcna1, Kcna2, Kcnab3*), whereas others were upregulated or flat in A1 and MG (*Kcna4, Kcnb1, Kcnk2, Kcnh1, Kcnt2, Kcnv1*). In addition, several of the upregulated SOC genes were expressed at only nominal levels in A1 or MG (*Kcn15, Kcns3, Kcnk5, Kcnj8*). These differences signify regional differences in the distribution of potassium channels between the SOC and auditory forebrain, and are also likely to reflect many other differences that we did not take time to compare here.

Finally, two studies profiled changes in gene expression after manipulations of activity in the central auditory pathways. Holt et al. (2005) used targeted microarrays to track transcript expression in the rat IC at 3, 21 and 90 days after bilateral deafening. Variable trajectories in expression were observed in GABA, glycine, glutamate, and serotonin receptor genes, among the many others profiled [35]. The expression of several genes increased after deafening (their nomenclature: *GluR2, GABA-A A1, GABA-A B2, GABA-A B3, 5HT2C*). By way of comparison with the present study (refer to Fig. 7), we found that *GluR2* (*Gria2*) was steadily upregulated after hearing onset in A1, but downregulated in the MG; *GABA-A A1* and *B2* (*Gabra1, Gabrb2*) had upward trajectories from P7 to adult in A1 and MG; *GABA-A B3* (*Gabrb3*) decreased in A1 and MG; and *5HT2C* (*Htr2c*) increased between P7 and P14, then declined in A1 and MG. Although auditory activity, per se, could be considered a common variable between studies, comparison of the just a portion of the findings highlights the complex and rather unpredictable regional differences that could also be associated with activity-dependent regulation of the same genes. Clarkson et al. (2012) used microarrays and qPCR to track gene expression in the IC after unilateral lesions of the auditory cortex (affecting the cortico-tectal projections to both hemispheres) [36]. Their dataset cannot be succinctly summarized, but a general trend relevant to this discussion was that genes related to neurotransmission and synaptic growth (among many others) were downregulated in the ipsilateral IC and upregulated contralaterally, presumably reflecting changes in the balance of excitation and inhibition. Thus, in both studies, transcript profiling was sensitive to changes induced by the manipulation in each of the brain regions studied. Although not yet applied in this manner, whole transcriptome sequencing would be expected to be at least as sensitive as microarrays to such changes, with the added advantage that the entire transcriptome can be profiled.

### Applications and future directions

RNAseq is a powerful tool for mRNA profiling and transcriptome analyses, with broad potential applicability in neurobiology [105]. Relatively small amounts of starting material (<10 ng) are sufficient to conduct whole transcriptome sequencing of discrete brain areas or cell populations. The reduction in sequencing costs, development of bioinformatics tools, and availability of genomic libraries add further to the attractiveness of this approach [106, 107]. As mentioned above, an important advantage of targeted profiling of selected genes using qPCR or microarrays is that data from the entire transcriptome is obtained. This removes limits on the identification of novel or unexpected changes, and broadens the scope of pathway analyses.

The dataset generated by this study comprises an extensive reference library that indexes the expression of any gene or gene family in the A1 and MG from P7 to adult. In addition to information about these structures during postnatal development, the dataset is also a rich source of information about mature animals. We envision several potential uses of this dataset by those interested in the structure and function of the auditory forebrain.

One application would be as a screening tool to guide hypothesis formation and streamline the design of neuroanatomical and neurophysiological studies [108]. The dataset provides *a priori* knowledge of the expression levels of genes and gene pathways that may be targeted for studies of expression *and* co-expression patterns in intact tissue sections. That is, what are the subpopulations of cells in A1 or MG that express the genes of interest, and to what extent are they colocalized, or not? We used this approach in our recent study of the vesicular transporters of glutamate and GABA in developing mouse auditory forebrain [53]. In addition to augmentation of anatomical libraries, that information could also be used to guide selection of transgenic models for neurophysiological and behavioral assays, and improve the specificity of optogenetic or pharmacological studies where a particular cell population or receptor type is targeted.

A second, and related, application is to provide a baseline for experimental studies (e.g., altered sound exposure during development, hearing loss, aging, other pathology) [109, 110, 35, 36]. Transcriptomic analyses of global or targeted gene expression are powerful means to identify genes that are changing the most (or the least). Relationships between members of different gene families and functional pathways can easily be extracted and incorporated into analyses of functional pathways and construction of models.

Finally, we also envision that transcriptome profiles could be used to conduct comparative genomic studies in other animal models, including humans. We have long been aware of species differences in gene and protein expression in the auditory pathways [111–113] – an observation that is in line with a growing number of studies in the brain [114–119, 72, 120]. Documentation of species differences is absolutely essential to make informed conclusions and predictions about the roles of particular genes, and we must be vigilant to consider such differences in the interpretation of and application of profiling data.

### Caveats and limitations

#### GSEA analysis

GSEA fostered the identification of GO categories and gene families that were enriched in each region of interest. A general advantage of this approach is that well-developed analytical tools are available to identify robust patterns in large datasets, such as those produced by RNAseq. A potential weakness is that important differences in the expression of some genes or entire gene families may be overlooked because they were ranked low by GSEA.

Application of GSEA to *GO categories* permits profiling of the expression patterns among genes that were grouped into functional categories based on published data. In that sense, they are a useful guide to the identification and exploration of pathways involved in a particular process. One disadvantage is that the underlying data may not apply to all regions of interest (i.e., auditory forebrain). That is, the data used to develop a GO category may be derived from a brain region or cell population for which transcriptome profiles differ substantially from the current region of interest. The differences observed between A1 and MG are examples of this consideration. A second limitation is that GO categories are often incomplete and subject to change as new data are obtained. In that sense, they are not invariant descriptors of the genes and regulatory factors that contribute to a given function. Finally, a limitation of all such analyses is that genes are often multifunctional and may appear in multiple GO categories.

Application of GSEA to *gene families* facilitates identification of novel patterns and relationships between genes in the same family or related families. One limitation is that co-expression or co-variation of genes within a family does not imply that a functionally-significant relationship exists between those genes. Most functional pathways involve interactions between genes across multiple families. Secondly, since the members of gene families often have different expression profiles, some genes with functional relevance may be missed in the categorical GSEA analysis.

Thus, application of GSEA is useful for identification of major trends, but is best used in combination with tools that enable construction of custom GO categories and pathway analyses.

#### Single-gene profiling

An important goal of the analyses that were employed in this study was to distill some of most important expression patterns identified at the global transcriptome level into manageable gene-level assemblages with functional relevance. Unfortunately, an exhaustive treatment of the interesting trends and relationships contained within this dataset are rather far beyond the scope of any single paper. Instead, we highlighted subsets of the data through analyses of selected genesets, along with single gene analyses to fill in the details. In so doing, many hundreds of genes with significant functionality were not profiled in the main body of the manuscript. Fortunately, information about any gene in the library can be easily extracted and plotted from the supplementary Tables or by using the Look-Up tool.

One proviso concerns the interpretation of gene expression magnitude (read counts) in such data. For example, if a gene has low or even undetectable expression in a sample, or if changes in expression are not significant, several explanations may apply [121]: (1) the gene is expressed at very low levels across all cells in the sample; (2) the gene is expressed at moderate to high levels, but in a small subpopulation of cells; and (3) the somata containing the transcripts are located outside the region of harvest (e.g., endogenous ligands, vesicular transporters). Conversely, if a gene is expressed at high levels, comparable questions about specificity also apply. For example, it may be that a gene is expressed at high levels only within certain cell subpopulations or anatomical subdivisions within the sample. Therefore, interpretation of all results must be scrutinized within a valid anatomical framework. That would include, but not be limited to, knowledge of the brain region, layer or nucleus, and specific cell types that express each gene [122, 71, 123], as well as their patterns of co-expression with other related genes.

A second limitation is that, in their present form, our data do not directly address the identity of the elements that are regulating or driving changes in gene expression within a region. Are genetic factors driving the formation of functional networks? Are the developing networks or external stimuli driving de novo gene expression adaptively? Does a statistically-significant change in expression have functional significance? The biological importance of a regional or age-related change in gene expression must be determined by other means. Therefore, the data contained in this manuscript may best be used to drive hypotheses and inform the design of experiments that can provide answers to these questions.

## Conclusions

Transcriptome profiling at the global, group, and single gene levels revealed that gene expression is in constant flux from P7 to adult in both A1 and MG. Globally, expression profiles are strongly clustered by brain region and age. The greatest changes in global gene expression occur between P7 and P14 – before and after the onset of hearing. Thereafter, differences between age groups declined with age, consistent with an increase in the stability of gene expression patterns with postnatal age. At the group level, functional GO categories and gene families were up- or down-regulated from P7 to adult in a manner consistent with their functional roles. Overall, genesets related to the establishment of brain structure (architecture) were down-regulated as the brain matured. In contrast, genesets involved with neuronal activity and plasticity were up-regulated, especially after the onset of hearing. At the single gene level, maturational profiles varied by brain region and age, and in a manner that was not always predicted by analyses at the group level. Although functional studies are lacking at present, the database generated by this study provides a foundation for the identification of pathways that operate specifically within the auditory forebrain.

### Availability of Supporting Data

The complete set of raw sequencing files is available from the National Center for Biotechnology Information (NCBI) database under accession number SRP053237 (http://www.ncbi.nlm.nih.gov/projects/geo/). All other supporting data are included in the Additional files section.

## Additional files

Additional file 1: Figure S1. Sample harvesting. Low magnification coronal images at the level of A1 and MG. (A) Gapdh in situ hybridization; (B) photograph of a frozen brain during harvesting of samples from A1 and MG for sequencing. The location of A1 within the auditory cortex (AC) is shown, along with a sketch of the 0.5 mm punch used to obtain samples. Note that the size and shape of the punch compresses tissue outside of the punched volume. The left MG has been circumscribed prior to extraction. Scale bars, 1 mm all panels. Additional file 2: Table S1. Population and quality control data for all A1 and MG samples obtained for total RNA sequencing. For each sample, the following information is provided: region of interest (ROI), age, sex, RNA integrity number (RIN), 28 s:18 s ratio, 260/280 ratio. Samples are arranged sequentially by an identifier that is used in all other tables. **Table S2.** Raw data quality control matrix. Contains information about the sequencing platform, total reads, and sample quality measures. **Table S3.** Quality control of alignments. For each sample, information about the sequencing platform and mapping details are listed. **Table S4.** Raw counts of all genes for the 48 samples sequenced. Additional file 3:
**Differential expression comparing A1 and MG at at P7, P14, P21, and Adult.** **Table S5.** Differential expression analyses comparing MG with A1 at P7 for all genes using DESeq2, EdgeR, and BaySeq. For each gene, the log2 fold-change, p-values, and rank are listed. The final column (All Rank) is the sum of rankings obtained by all three methods. Gene listing is given in. **Table S6.** Differential expression analyses comparing MG with A1 at P14 for all genes using DESeq2, EdgeR, and BaySeq. For each gene, the log2 fold-change, p-values, and rank are listed. The final column (All Rank) is the sum of rankings obtained by all three methods (lowest number = highest rank). Gene listing is ordered from highest to lowest rank. **Table S7.** Differential expression analyses comparing MG with A1 at P21 for all genes using DESeq2, EdgeR, and BaySeq. For each gene, the log2 fold-change, p-values, and rank are listed. The final column (All Rank) is the sum of rankings obtained by all three methods (lowest number = highest rank). Gene listing is ordered from highest to lowest rank. **Table S8.** Differential expression analyses comparing MG with A1 in adult animals for all genes using DESeq2, EdgeR, and BaySeq. For each gene, the log2 fold-change, p-values, and rank are listed. The final column (All Rank) is the sum of rankings obtained by all three methods (lowest number = highest rank). Gene listing is ordered from highest to lowest rank. Additional file 4:
**Differential expression in the MG for age group comparisons with P7 (P7-Adult, P7-P14, P7-P21).** **Table S9.** Differential expression analyses in the MG comparing P7 with Adult using DESeq2, EdgeR, and BaySeq. For each gene, the log2 fold-change, p-values, and rank are listed. The final column (All Rank) is the sum of rankings obtained by all three methods (lowest number = highest rank). Gene listing is ordered from highest to lowest rank. **Table S10.** Differential expression analyses in the MG comparing P7 with P14 using DESeq2, EdgeR, and BaySeq. For each gene, the log2 fold-change, p-values, and rank are listed. The final column (All Rank) is the sum of rankings obtained by all three methods (lowest number = highest rank). Gene listing is ordered from highest to lowest rank. **Table S11.** Differential expression analyses in the MG comparing P7 with P21 using DESeq2, EdgeR, and BaySeq. For each gene, the log2 fold-change, p-values, and rank are listed. The final column (All Rank) is the sum of rankings obtained by all three methods (lowest number = highest rank). Gene listing is ordered from highest to lowest rank. Additional file 5:
**Differential expression in the MG for age group comparisons between P14, P21 and Adult (P14-P21, P14-Adult, P21-Adult).** **Table S12.** Differential expression analyses in the MG comparing P14 with P21 using DESeq2, EdgeR, and BaySeq. For each gene, the log2 fold-change, p-values, and rank are listed. The final column (All Rank) is the sum of rankings obtained by all three methods (lowest number = highest rank). Gene listing is ordered from highest to lowest rank. **Table S13.** Differential expression analyses in the MG comparing P14 with Adult using DESeq2, EdgeR, and BaySeq. For each gene, the log2 fold-change, p-values, and rank are listed. The final column (All Rank) is the sum of rankings obtained by all three methods (lowest number = highest rank). Gene listing is ordered from highest to lowest rank. **Table S14.** Differential expression analyses in the MG comparing P21 with Adult using DESeq2, EdgeR, and BaySeq. For each gene, the log2 fold-change, p-values, and rank are listed. The final column (All Rank) is the sum of rankings obtained by all three methods (lowest number = highest rank). Gene listing is ordered from highest to lowest rank. Additional file 6:
**Differential expression in A1 for age group comparisons with P7 (P7-Adult, P7-P14, P7-P21).** **Table S15.** Differential expression analyses in A1 comparing P7 with Adult using DESeq2, EdgeR, and BaySeq. For each gene, the log2 fold-change, p-values, and rank are listed. The final column (All Rank) is the sum of rankings obtained by all three methods (lowest number = highest rank). Gene listing is ordered from highest to lowest rank. **Table S16.** Differential expression analyses in A1 comparing P7 with P14 using DESeq2, EdgeR, and BaySeq. For each gene, the log2 fold-change, p-values, and rank are listed. The final column (All Rank) is the sum of rankings obtained by all three methods (lowest number = highest rank). Gene listing is ordered from highest to lowest rank. **Table S17.** Differential expression analyses in A1 comparing P7 with P21 using DESeq2, EdgeR, and BaySeq. For each gene, the log2 fold-change, p-values, and rank are listed. The final column (All Rank) is the sum of rankings obtained by all three methods (lowest number = highest rank). Gene listing is ordered from highest to lowest rank. Additional file 7:
**Differential expression in A1 for age group comparisons between P14, P21 and Adult (P14-P21, P14-Adult, P21-Adult).** **Table S18.** Differential expression analyses in A1 comparing P14 with P21 using DESeq2, EdgeR, and BaySeq. For each gene, the log2 fold-change, p-values, and rank are listed. The final column (All Rank) is the sum of rankings obtained by all three methods (lowest number = highest rank). Gene listing is ordered from highest to lowest rank. **Table S19.** Differential expression analyses in A1 comparing P14 with Adult using DESeq2, EdgeR, and BaySeq. For each gene, the log2 fold-change, p-values, and rank are listed. The final column (All Rank) is the sum of rankings obtained by all three methods (lowest number = highest rank). Gene listing is ordered from highest to lowest rank. **Table S20.** Differential expression analyses in A1 comparing P21 with Adult using DESeq2, EdgeR, and BaySeq. For each gene, the log2 fold-change, p-values, and rank are listed. The final column (All Rank) is the sum of rankings obtained by all three methods (lowest number = highest rank). Gene listing is ordered from highest to lowest rank. Additional file 8: Figure S2. Comparison of sequencing with in situ hybridization. (*Top*) In situ hybridization of *Slc32a1, Slc17a6, Slc17a7* from P7 to adult in A1 and MG. Coronal sections. Roman numerals indicate cortical layers. Abbrevations: d, dorsal division of MG; v, ventral division of MG; sp, subplate layer. Scale bars, 250 μm all panels. (*Bottom*) Normalized counts from RNAseq are compared to expression levels derived from quantitative densitometry of colorimetric in situ hybridization (ISH) assays performed at each maturational age (P7, P14, P21, Adult). Results are plotted separately for MG and A1. The housekeeping gene, *Gapdh*, had a flat maturational trajectory for both RNAseq and ISH, and was used for normalization of the ISH grayscale intensity measurements. Additional file 9: Table S21. Read counts for genes in all HUGO gene families. Normalized read counts for 19,826 genes in all HUGO gene families currently listed in that resource, listed alphabetically by gene family. Source: HUGO Gene Families database, HUGO Gene Nomenclature Committee at the European Bioinformatics Institute (http://www.genenames.org). **Table S22.** Gene set enrichment analysis of 111 HUGO gene families. Genesets are listed by functional group and family (from Table S21). For each geneset, the number of genes (Size), false discovery rate (FDR q-value), and direction of expression (up, down) are listed. Shading denotes gene families enriched above the 25 % FDR cutoff. **Table S23.** Normalized read counts for genes in selected gene ontology (GO) categories from the MGI database. Genes are arranged alphabetically by GO category, along with gene symbols, names, and annotations. Source: Mouse Genome Informatics (MGI) Gene Ontology Browser, Jackson Laboratories (www.informatics.jax.org). **Table S24.** Gene set enrichment analysis of the 51 gene ontology categories. Genesets are listed by functional group and GO designation (from Table S23). For each geneset, the number of genes (Size), false discovery rate (FDR q-value), and direction of expression (up, down) are listed. Shading denotes categories enriched above 25 % FDR cutoff. Additional file 10: Table S25. Gene families database. Normalized read counts and plots of ~4700 genes in 237 gene families from the HUGO database. Gene families are grouped by 20 functional categories arranged in tabs. For each family, normalized read counts (mean and % of maximum) are provided for A1 and MG as a function of postnatal age to permit rapid screening of the relationships between genes by brain region and age. Additional file 11:
**Look-up and plotting tool.** This tool supports automated extraction of normalized counts for A1 and MG, and generates a plot of the counts and correlation matrix comparing maturational trajectories between each age group. A list of up to 25 genes can be entered into Column A. Use standard gene names only. Not case sensitive, but omit empty spaces. The normalized counts will populate in columns B – AU, and the data plotted below (profile plots, correlation matrices). Erase or overwrite gene names to generate a new listing. Counts are extracted from the Normalized Reads tab. This tab is locked and should not be edited.
