# Supplementary figures and images for: Transcriptional maturation of the mouse auditory forebrain
Source: BMC Genomics. 2015 Aug 14;16(1):606. doi: 10.1186/s12864-015-1709-8 (PMC4536593; doi:10.1186/s12864-015-1709-8)

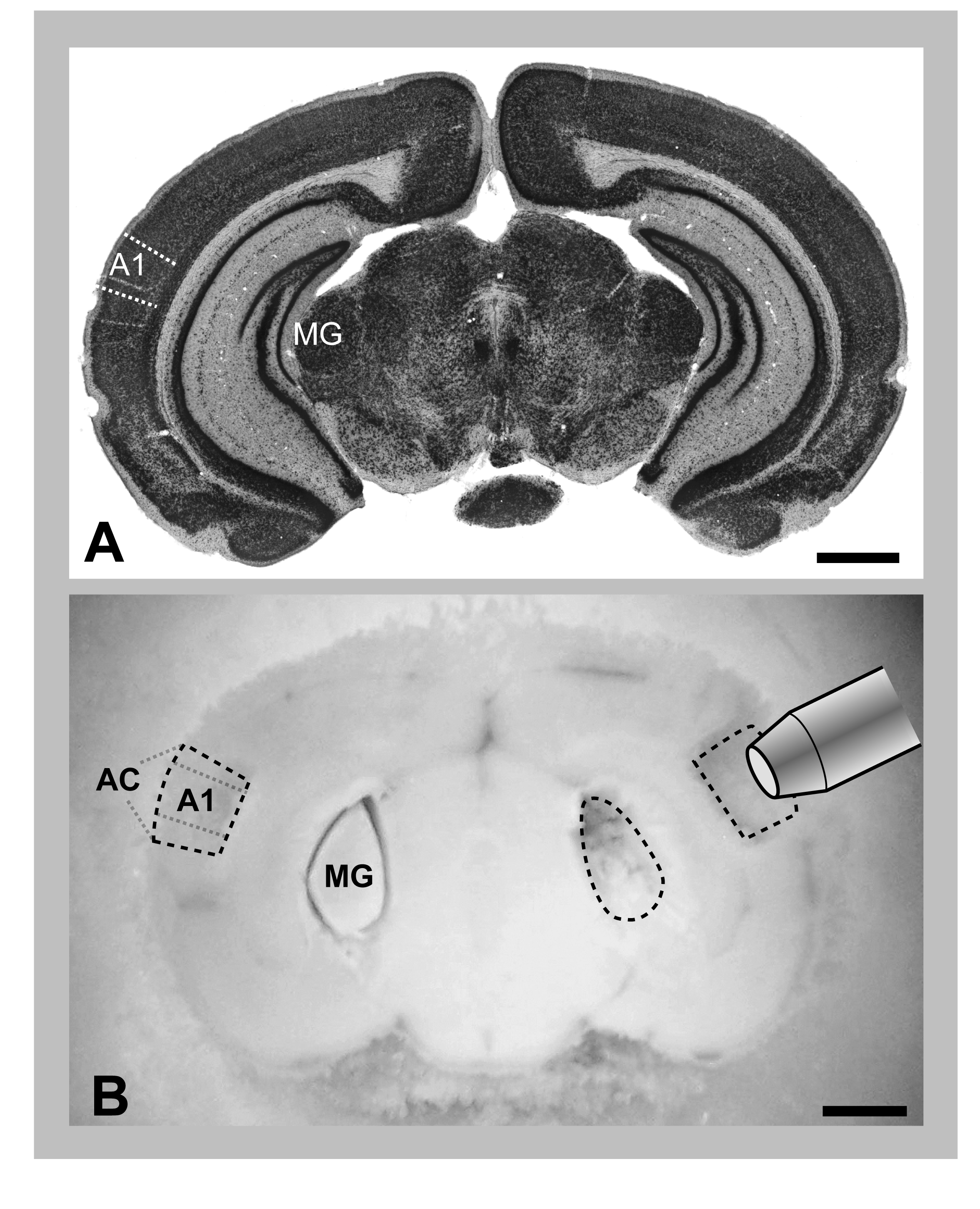

Supplement: Additional file 1: Figure S1. — Sample harvesting. Low magnification coronal images at the level of A1 and MG. (A) Gapdh in situ hybridization; (B) photograph of a frozen brain during harvesting of samples from A1 and MG for sequencing. The location of A1 within the auditory cortex (AC) is shown, along with a sketch of the 0.5 mm punch used to obtain samples. Note that the size and shape of the punch compresses tissue outside of the punched volume. The left MG has been circumscribed prior to extraction. Scale bars, 1 mm all panels. [file 12864_2015_1709_MOESM1_ESM.tiff]

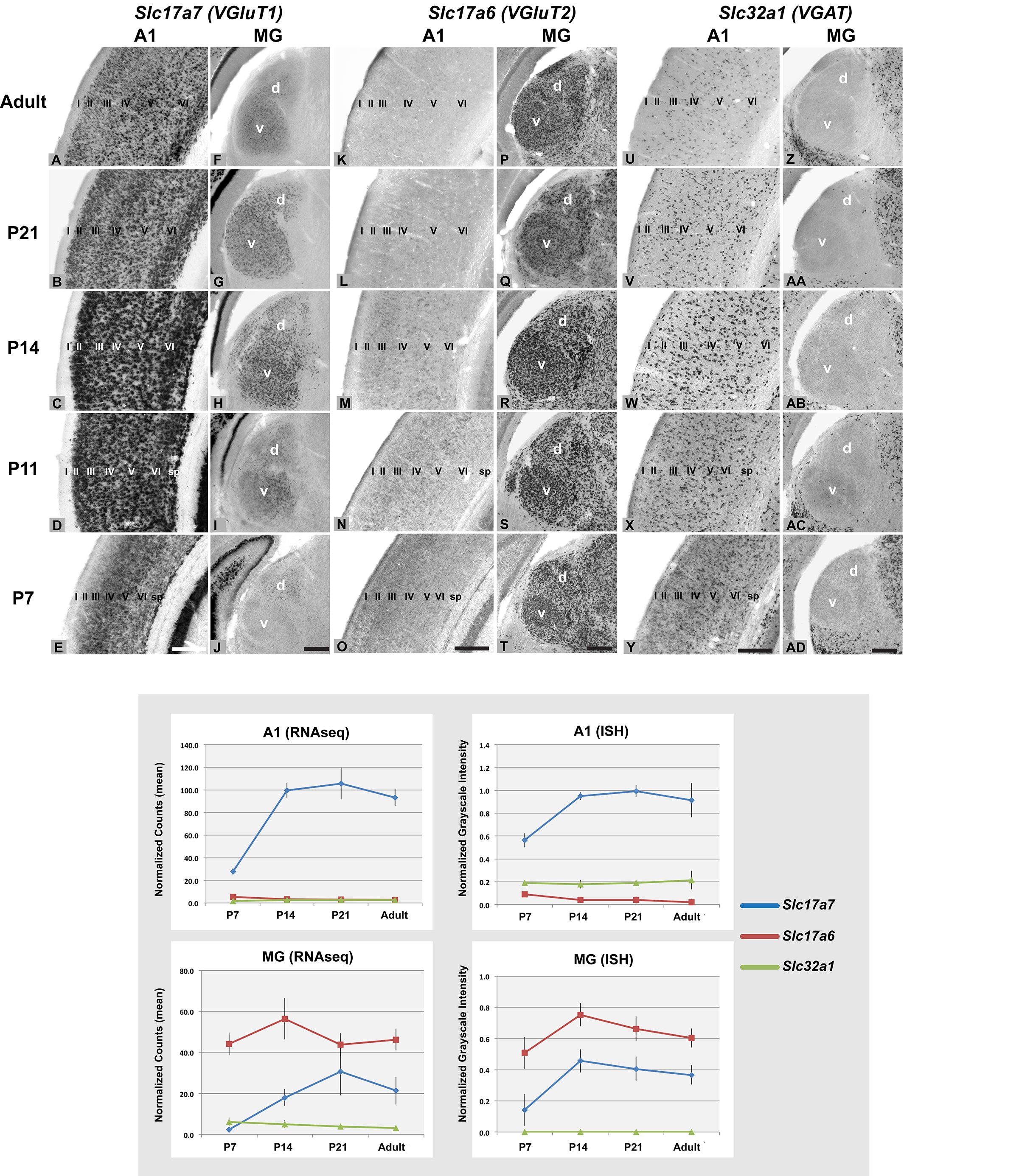

Supplement: Additional file 8: Figure S2. — Comparison of sequencing with in situ hybridization. (Top) In situ hybridization of Slc32a1, Slc17a6, Slc17a7 from P7 to adult in A1 and MG. Coronal sections. Roman numerals indicate cortical layers. Abbrevations: d, dorsal division of MG; v, ventral division of MG; sp, subplate layer. Scale bars, 250 μm all panels. (Bottom) Normalized counts from RNAseq are compared to expression levels derived from quantitative densitometry of colorimetric in situ hybridization (ISH) assays performed at each maturational age (P7, P14, P21, Adult). Results are plotted separately for MG and A1. The housekeeping gene, Gapdh, had a flat maturational trajectory for both RNAseq and ISH, and was used for normalization of the ISH grayscale intensity measurements. [file 12864_2015_1709_MOESM8_ESM.tiff]
